# Charge‐Driven Self‐Assembly of Cholesterol Surfactants into Biofunctional Nanodiscs with Antiviral Activity

**DOI:** 10.1002/anie.202516207

**Published:** 2025-11-13

**Authors:** Yanping Long, Seyyed Mohammad Mousavifard, Xianfeng He, Roland R. Netz, Hesam Makki, Mathias Dimde, Chuanxiong Nie, Abhishek K. Singh, Rainer Haag

**Affiliations:** ^1^ Institute of Chemistry and Biochemistry Freie Universität Berlin Takustrasse 3 14195 Berlin Germany; ^2^ Forschungszentrum für Elektronenmikroskopie Core‐Facility BioSupraMol Institute of Chemistry and Biochemistry Freie Universität Berlin Fabeckstraße 36a 14195 Berlin Germany; ^3^ Department of Chemical Engineering University of Bath Bath BA2 7AY UK; ^4^ Department of Polymer and Color Engineering Amirkabir University of Technology 424 Hafez Tehran Iran; ^5^ Fachbereich Physik Freie Universität Berlin Arnimallee 14 14195 Berlin Germany

**Keywords:** Antiviral, Cholesterol, Nanodiscs, Oligo‐glycerol Surfactants, Vesicles

## Abstract

Self‐assembly of lipid structures derived from amphiphilic molecules plays a crucial role in the development of biomimetic systems. Here we report a modular synthetic strategy for developing cholesteryl‐oligo‐glycerol‐based surfactants with tunable head group functionalities ranging from nonionic to anionic. This approach enables the systematic incorporation of functional groups and thus precise control of surface charge and hydrophilicity. To investigate the influence of multivalent charges on supermolecular‐assembly behavior, we compared three structurally cholesterol (CL) related surfactants: CL‐4S, with four sulfate groups, CL‐1S, with a single sulfate group, and CL‐4OH, a nonionic analog with four hydroxyl groups. We then incorporated these surfactants into lipid bilayers of 1,2‐dimyristoyl‐sn‐glycero‐3‐phosphocholine (DMPC) and cholesterol (CL) to study their behavior in membrane‐like environments. Experimental, simulation, and theoretical studies demonstrated that the CL‐4S formulation was able to convert lipid vesicles into nanodiscs, unlike CL‐1S and CL‐4OH, demonstrating the importance of adequate charges in supramolecular transition. Furthermore, both 1S‐Vesicles (CL‐1S based sulfated vesicles) and 4S‐Nanodiscs (CL‐4S based sulfated nanodiscs) showed inhibitory activity against herpes simplex virus‐1 (HSV‐1), indicating the potential of this multivalent supramolecular platform for antiviral applications.

## Graphical abstract

A modular synthesis strategy for cholesterol‐oligo‐glycerol‐based surfactants with tunable nonionic to anionic head groups is reported. The precise control of multivalent surface charges directs self‐assembly and transforms cholesterol vesicles into nanodiscs. Structurally optimized sulfated nanodiscs exhibit significant antiviral activity against HSV‐1. This approach emphasizes the therapeutic potential of charge‐directed supramolecular lipid nanostructures.

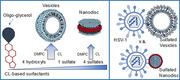

## Introduction

Self‐assembly, a fundamental process in nature, plays a pivotal role in biological systems by enabling the formation of complex and organized structures.^[^ ^1^, ^2^ ^]^ One of the best‐known examples is self‐assembly of phospholipids into bilayers in cellular systems, a process that creates selective barriers essential for cell membrane structure and functions.^[^ ^3^, ^4^ ^]^ Inspired by natural self‐assembly and enabled by advances in material science, artificial supramolecular systems, have been developed to produce well‐defined, dynamic, and functional nanostructures. Among these, artificial lipid bilayer nanodiscs are characterized by precise diameter, stability in solution and mimicry of natural lipid bilayers. From an application perspective, nanodiscs offer a native‐like environment for membrane proteins, thereby enabling the development of therapeutic delivery platforms.^[^ ^5^, ^6^, ^7^, ^8^ ^]^ Surfactant‐based lipid bilayer nanodiscs are of particular interest because, as compared to lipid bilayer nanodiscs formed by membrane scaffold proteins or peptides, they are inexpensive, easy to produce, chemically stable. Furthermore, their functional characteristics can be adjusted by modifying the surfactants’ structures.^[^ ^9^, ^10^ ^]^

Structurally, the surfactant component plays an important role in self‐assembly and functionality; a balance between hydrophobic and hydrophilic interactions is extremely important.^[^ ^11^ ^]^ In the nanodiscs field, given styrene‐co‐maleic acid and its derivations restricts few biophysical techniques, more researchers have turned to styrene‐free systems. For example, Ramamoorthy's group developed an amphiphatic polymer composed of hydrophobic butyl methacrylate and cationic methacroylcholine chloride; their work demonstrated that fragmentation of the vesicular membrane can only occur in the case of moderately hydrophobic polymers.^[^ ^12^ ^]^ Furthermore, surfactants based on cholesterol have attracted growing interest because of its high compatibility and stability, and because it is an irreplaceable component in natural cell membrane. For example, Li's group designed and synthesized four kinds of cholesterol‐analog surfactants, finding that these surfactant‐including lipid particles showed excellent stability, mRNA encapsulation efficiency, and selective targeting delivery toward the spleen and lungs.^[^ ^13^ ^]^ On the other side, the hydrophilic head groups, compared with traditional hydropathic systems, oligo‐glycerol‐based surfactants offer advantages due to their multivalency, biocompatibility, and post‐functional tunability. Especially they can be functionalized with charged functional groups (e.g., sulfonates, carboxylates, and ammonium) and enable more diverse studies.^[^ ^14^, ^15^, ^16^, ^17^ ^]^ Despite this significant potential, charged nanodiscs composed of a cholesterol moiety and a charged moiety have rarely been reported. Additionally, these nanodiscs’ nature as supramolecular polymers, characterized by non‐covalent interactions, imparts dynamic structural integrity and compositional flexibility. Such properties are advantageous for biomedical applications, including drug delivery, membrane protein stabilization,^[^ ^8^, ^18^, ^19^ ^]^ and potential application in antiviral strategies.

**Figure 1:**
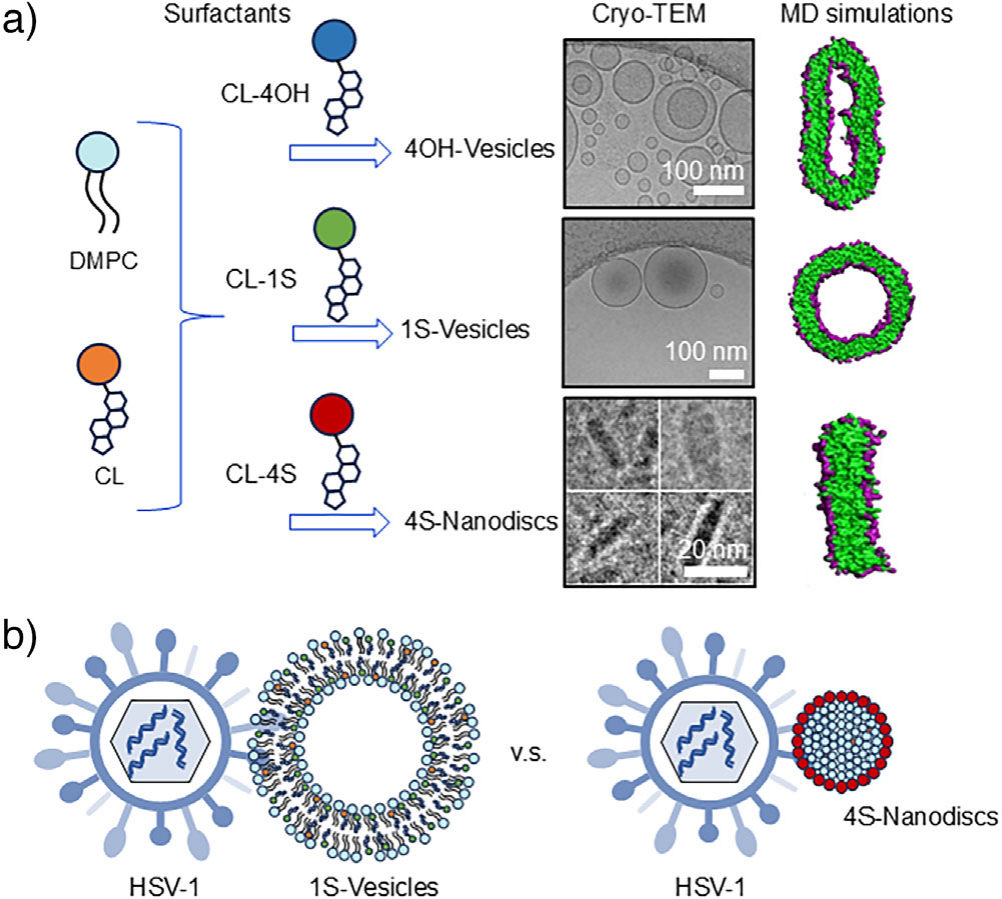
a) Formulation of 1S‐Vesicles (DMPC/CL‐1S/CL), 4OH‐vesicles (DMPC/CL‐4OH/CL), and 4S‐Nanodiscs (DMPC/CL‐4S/CL). b) Binding interaction of HSV‐1/1S‐Vesicles and HSV‐1/4S‐Nanodiscs were showcased. For the abbreviations, DMPC: 1,2‐dimyristoyl‐sn‐glycero‐3‐phosphocholine; CL: cholesterol; CL‐1S: cholesterol‐monosulfate group; CL‐4OH: cholesterol‐tetrahydroxyl groups; CL‐4S: cholesterol‐tetrasulfate groups; HSV‐1: herpes simplex virus‐1; MD: molecular dynamics.

In this study, we investigate the relationship between the rational design of cholesterol‐based surfactants and their emerging supramolecular behavior, with particular emphasis on their potential as functional, non‐covalent supramolecular polymers. Using a modular synthetic approach, we constructed amphiphilic cholesterol‐based surfactants containing either tetra‐sulfate (CL‐4S) or tetra‐hydroxyl (CL‐4OH) head groups, allowing a controlled comparison of charged and neutral surface functions. To compare the number of charges, we used commercially available cholesterol with one sulfate group (CL‐1S). These surfactants were incorporated into lipid bilayer systems of DMPC and CL to investigate how the charge of the head group affects self‐assembly. We observed distinct differences in morphology depending on the identity of the headgroup, suggesting that multivalent charge interactions play a key role in shaping nanoscale self‐assembly processes. In addition to the structural findings and multiscale modeling by atomistic and coarse‐grained simulations as well as continuum theory, we assessed the biological relevance of these arrangements and investigated their potential to disrupt viral infectivity, thus establishing a link between molecular design and functional application in antiviral nanomaterials (Figure 1).

## Results and Discussion

### Synthesis of Cholesterol‐Based Surfactants

**Scheme 1:**
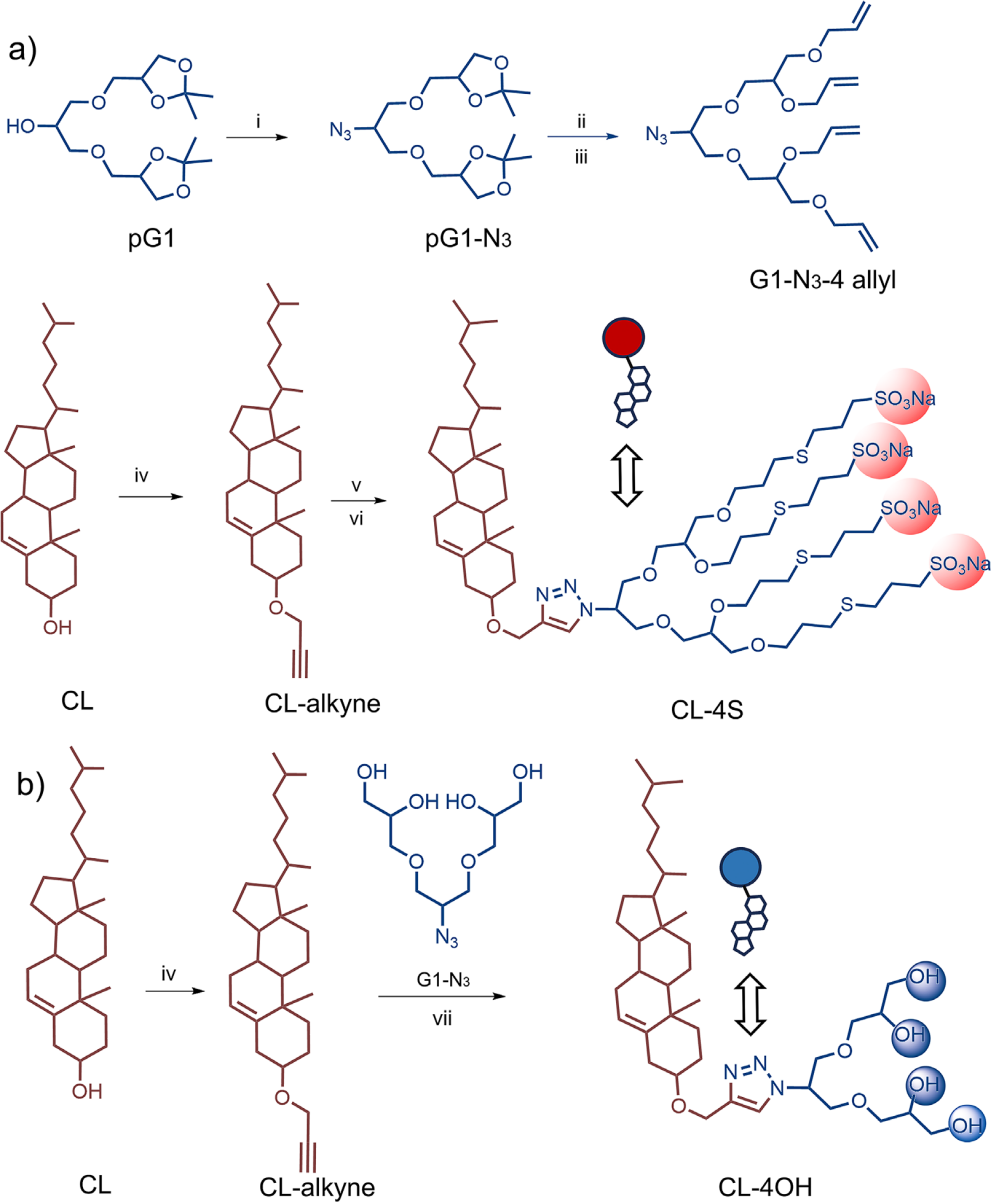
a) Synthesis of CL‐4S. (i) MsCl/TEA, DCM, 0 °C, 2 h; NaN_3_, dry DMF, 80 °C, 48 h. (ii) Dowex‐50, MeOH, 50 °C, 24 h. (iii) NaH, Allyl bromide, dry DMF, 50 °C, 24 h. (iv) Propargyl bromide, NaH, THF, 50 °C, 48 h. (v) G1‐N_3_‐4 allyl, Cu (I) acetate, THF, 50 °C, 24 h. (vi) MPS, Methanol/DMF, r.t., 6 h, under 365 nm LED lamp. b) Synthesis of CL‐4OH. (vii) Cu (I) acetate, THF, 50 °C, 24 h.

In the design of amphiphilic molecules, the selection of the monomer is a key decision, as its chemical structure directly influences the surfactant's self‐assembly behavior, molecular packing, and interaction with the environment. Tailoring features such as head group polarity, the spacer flexibility and the hydrophobic tail length allows precise control over the resulting supramolecular architecture and its suitability for targeted applications.^[^ ^20^, ^21^, ^22^, ^23^ ^]^ We prepared two kinds of cholesterol‐based surfactants synthesized via orthogonal click chemistry: one with a head group of four sulfate groups (CL‐4S) and the other with a head group of four hydroxyl groups (CL‐4OH). In the first step of synthesis, protected Oligo‐glycerol (G1) azide (N_3_) was produced through mesylating followed by azidation, and these structures were characterized by ^1^H NMR in Figure S1–S3.^[^ ^15^, ^24^, ^25^, ^26^, ^27^ ^]^ Then protected G1‐N_3_ was subjected to deprotection followed by allylation to produce G1‐N_3_‐4 allyl. The G1‐N_3_‐4 allyl were fully characterized by ^1^H NMR and mass spectrometry, with the appearance of the allyl protons at 5.18–6.03 ppm as well as the peak at 448.2 *m/z* shown in Figure S4 & S5. On the other hand, alkylation of CL with propargyl bromide leads to CL‐alkyne, which subsequently reacted with G1‐N_3_‐4 allyl through a Cu‐catalysed “click reaction” that produced CL‐4 allyl. Finally, the thiol‐ene reaction between CL‐4 allyl and sodium‐3‐mercapto‐1‐propanesulfonate (MPS) generated the CL‐4S surfactant (Figures S6–S9). In Figure S9, the intensity at 1538.5 *m/z* in mass spectra suggested the successful thiol‐ene reaction and the synthesis of our desired product (CL‐4S) (Scheme 1a). Meanwhile, two steps were required to obtain CL‐4OH. At first, the protected G1‐N_3_ was deprotected using Dowex 50W‐X8 to release free hydroxyl groups. Further, the deprotected G1‐N_3_ clicked with CL‐alkyne in the presence of Cu (I) acetate as catalyst. The CL‐4OH structure was confirmed by a prominent peak at 712.5 *m/z* in mass spectra (Scheme 1b and Figure S10).

**Figure 2:**
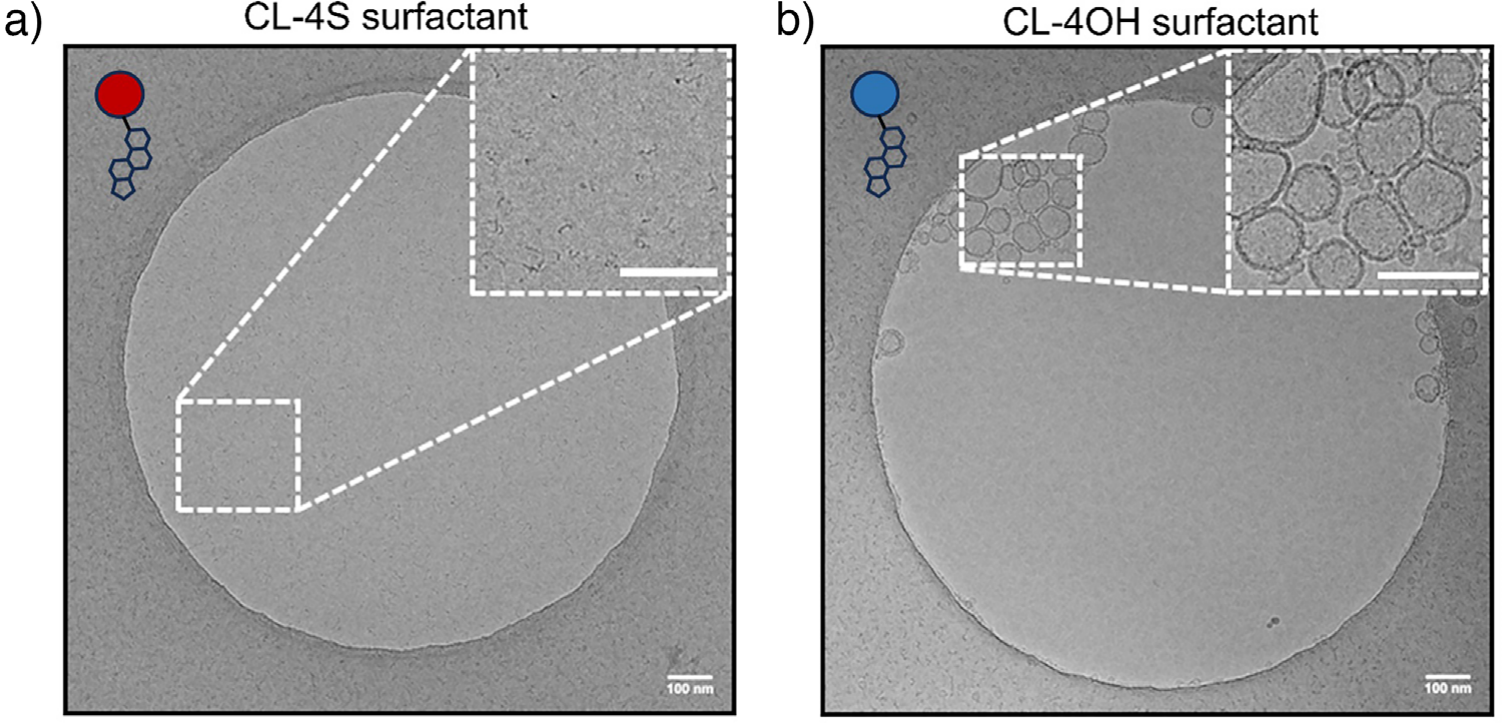
Cryo‐TEM image of a) CL‐4S and b) CL‐4OH surfactants in water. A selected area in Cryo‐TEM images is shown to reveal the worm‐like micelles of CL‐4S surfactant and vesicular morphology of CL‐4OH surfactant. The scale bar in images is 100 nm.

We then used cryo‐transmission electron microscopy (Cryo‐TEM) to perform further physicochemical characterizations of these two novel fabricated surfactants; we also measured critical micelle concentration (CMC) by fluorescence spectroscopy using Nile Red as a probe (Figure 2 and Figures S11, S12). Surprisingly, CL‐4S displayed worm‐like micelles, while CL‐4OH showed vesicular morphology. Further, the CMC of CL‐4S was measured at 0.55 mM. In addition to the synthetically produced CL‐4S and CL‐4OH, a commercially available cholesterol derivative with a single sulfate group (CL‐1S) was used for comparison in order to evaluate the supramolecular behavior toward DMPC/CL. The purpose of this comparison was to evaluate the effect of introducing multivalent oligo‐glycerol head groups with four sulfates.

### Self‐Assembly of Nanostructures

**Figure 3:**
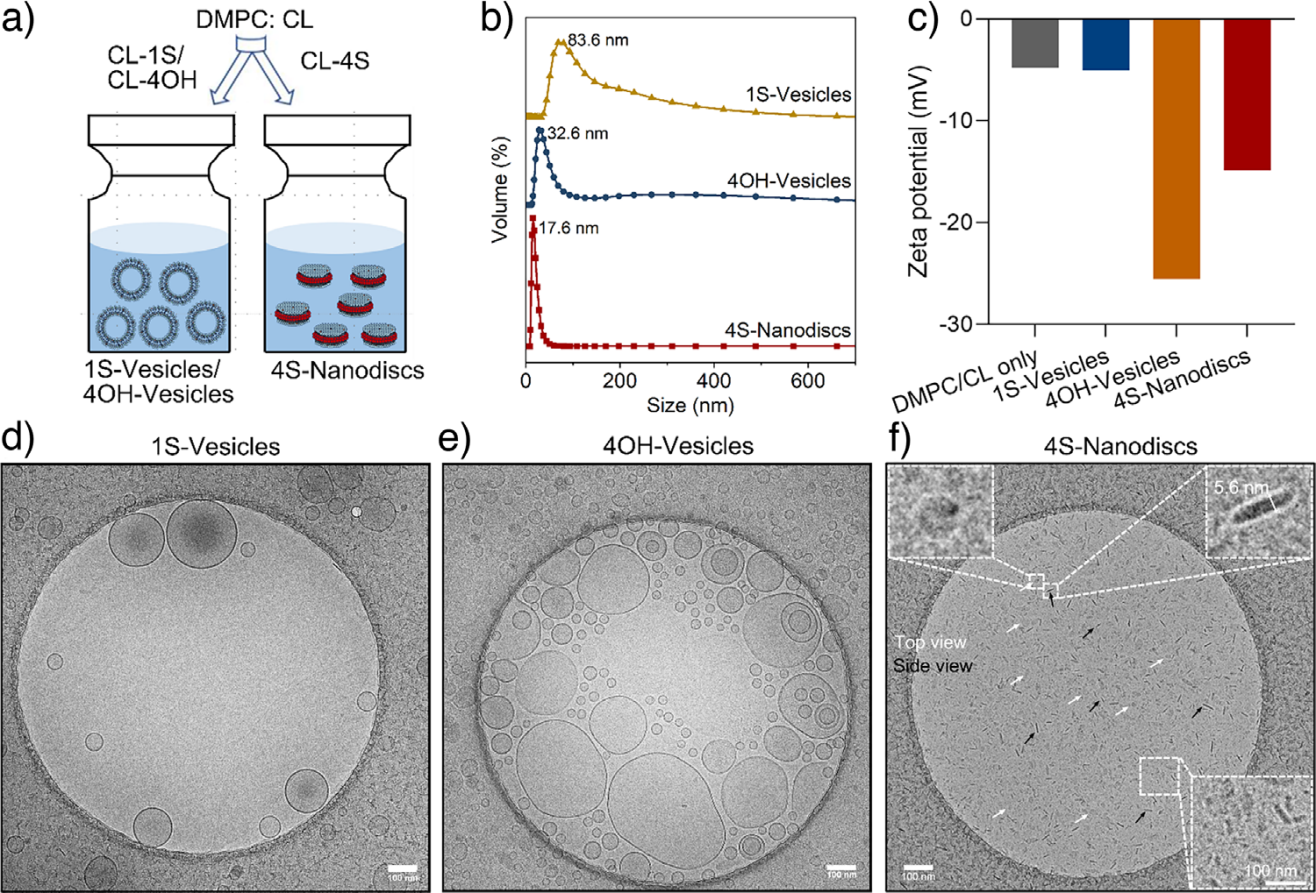
a) Schematic illustration of the formulation of 1S‐Vesicles, 4OH‐Vesicles and 4S‐Nanodiscs. b) DLS size distribution, and c) Zeta potential of various vesicles/nanodiscs. d), e), f) Cryo‐TEM images of 1S‐Vesicles, 4OH‐Vesicles, and 4S‐Nanodiscs. Scale bar=100 nm.

The self‐assembly behavior of the synthesized cholesterol‐based surfactants was studied by incorporating CL‐1S, CL‐4OH, and CL‐4S into lipid bilayers composed of DMPC and CL via the thin‐film hydration method (Figure 3a, details in Method 1.4). The obtained supramolecular assemblies were characterized using dynamic light scattering (DLS), Cryo‐TEM, and zeta potential analysis.

Cryo‐TEM showed that the incorporation of CL‐1S or CL‐4OH did not change the morphology of the nanostructure, which maintained a vesicle structure (Figure 3d,e). However, incorporating CL‐4S resulted in a distinct nanodiscs morphology measured at 5.6 nm in thickness (Figure 3f and Figure S13), consistent with the thickness of a single DMPC lipid bilayer,^[^ ^12^, ^28^, ^29^ ^]^ shows that bilayer integrity was retained even as the disc shape was adopted. DLS results (Figure 3b) confirm this observation by showing a significantly smaller and more homogeneous size distribution for CL‐4S‐containing systems as compared to vesicle‐based ones.

Zeta potential results (presented in Figure 3c) further supported the successful incorporation of surfactants, showing very low and almost identical charges on the surfaces of DMPC/CL only and the 4OH‐vesicles (−4.79 ± 0.11 and −5.04 ± 0.47 mV, respectively), while 1S‐vesicles displayed significant negative charge (−25.52 ± 1.74 mV), attributed to the sulfate group in CL‐1S. Meanwhile, the 4S‐Nanodiscs carrying sulfate groups also exhibited a lower negative charge (−14.85 ± 2.30 mV). These disparities in surface charge presumably derives from the surface area difference and curvature effects.^[^ ^30^, ^31^ ^]^

### Charge and Concentration Dependent Nanodiscs Formation

**Figure 4:**
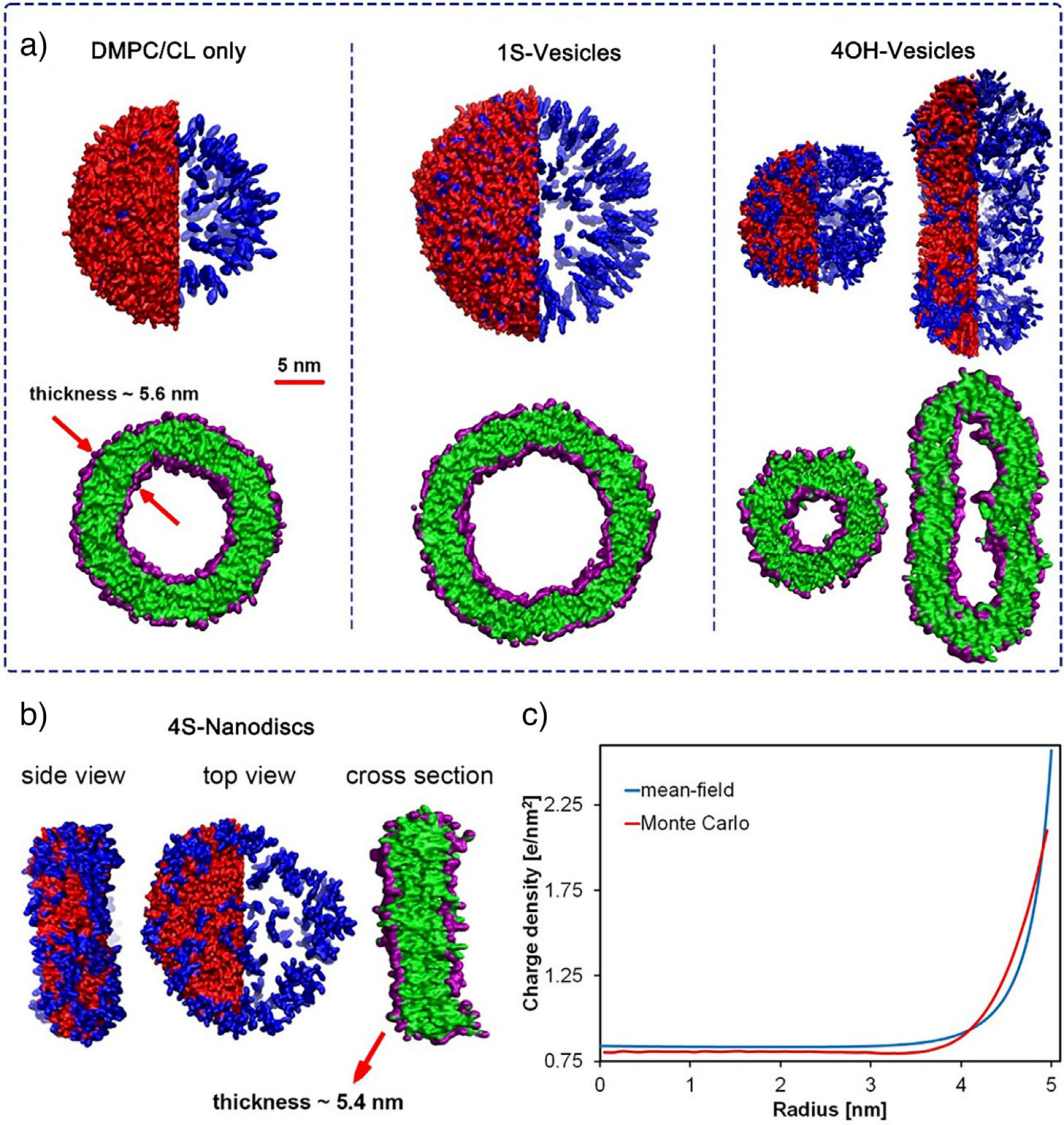
Simulation and continuum modelling results. a) Schematic representation of assemblies formed in different systems containing various cholesterol‐based surfactants. The top images show the side view of vesicles, in which red and blue regions represent DMPC and cholesterol‐based components (CL, CL‐1S, and CL‐4OH), respectively. For a better illustration, the DMPC‐containing regions are shown only in half of the assemblies to allow a clearer view of cholesterol distribution. The bottom images show cross‐sections of the corresponding assemblies, in which green and purple regions represent hydrophobic and hydrophilic beads. The thickness and scale bar represented for the DMPC/CL‐only system refers to all systems. b) Detailed structure of a simulated 4S‐nanodiscs. The colour representation is similar to part a): red and blue regions represent DMPC and CL‐4S, while green and purple regions represent hydrophobic and hydrophilic of all surfactants. c) Density distribution of mobile charges in a 2D disc of 10 nm in diameter calculated via continuum mean‐field theory and Monte Carlo simulations for an electrolyte concentration of *c*=100 mM (details in the section 3.2 including Figures S23–S25).

To further investigate the mechanisms responsible for nanostructure formation, we used atomistic and coarse‐grained molecular dynamics (MD) simulations as well as continuum mean‐field approaches and experimental analyses to explore the morphology transitions caused by charge and concentration variations. MD simulations captured the self‐assembly of DMPC/CL/cholesterol‐based surfactants consistent with experimental observations, in which CL‐1S and CL‐4OH form vesicles (CL‐1S and CL‐4OH: Figure 4a), while only CL‐4S can induce disc‐shaped morphology (Figure 4b). Moreover, all the simulated self‐assembled nano‐particles, i.e., those including DMPC/CL only, 1S‐, 4OH‐vesicles, as well as 4S‐nanodiscs, formed a bilayer of approximately 5.6 nm (Figure 4a,b) in agreement with Cryo‐TEM result (Figure S13). Furthermore, MD simulations and DLS analysis show unchanged vesicle morphology even under increased concentration of CL‐1S (Figures S14 and S22).

Enlarged snapshots from MD simulations of the 4S‐nanodiscs reveal a non‐uniform distribution of the highly charged CL‐4S surfactants, which tend to accumulate at the rims of the nanodiscs as opposed to the centers (Figure 4b). To further investigate the underlying mechanisms of this behavior, we used a continuum mean‐field and Monte Carlo approach of a highly coarse‐grained model of mobile charges interacting via screened Deby‐Hückel electrostatic interaction to calculate the charge distribution on a 2D disc with dimensions comparable to those observed in both the MD simulations and Cryo‐TEM –, i.e., approximately 10 nm in diameter (see Figure 4c and Supporting Information Section 3.2). This analysis also shows elevated local charge density at the disc periphery (Figure 4c), suggesting that CL‐4S behaves similarly to detergents, which tend to accumulate at bilayer edges and promote membrane destabilization.^[^ ^32^, ^33^ ^]^ The incorporation of CL‐4S into the lipid bilayer induces localized charge clustering, promoting nanodiscs formation by destabilizing bilayer growth. This destabilization likely arises from strong electrostatic repulsion between small, highly charged nanodiscs, which prevents these discs from colliding further and growing into vesicles. In contrast, incorporating low‐charged or neutral surfactants (e.g., CL‐1S, CL‐4OH), even at elevated concentrations, results in vesicle formation, indicating their limited capacity to disrupt bilayer structure in the manner required for nanodiscs stabilization. To further clarify this behavior, a video clip of the trajectory for formation of DMPC/CL‐vesicle is attached as Supporting Information (Video S1).

### Nanodiscs Self‐Assembly Process

**Figure 5:**
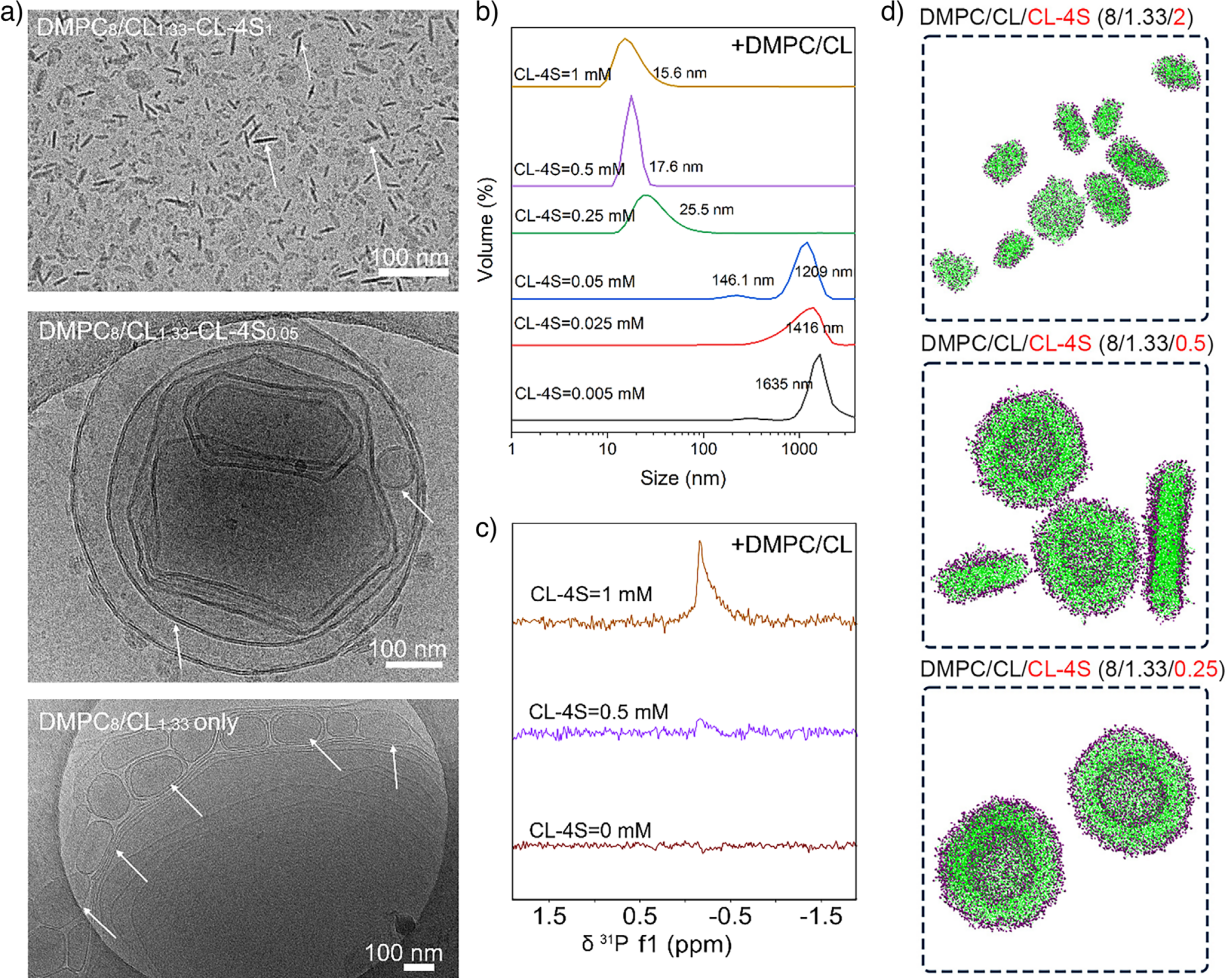
a) Cryo‐TEM images of DMPC/CL (8/1.33 mM) system with various CL‐4S amphiphile concentrations (0 mM (bottom), 0.05 mM, 1 mM (top)). Scale bar=100 nm. b) DLS profile of DMPC/CL (8/1.33 mM) system with various concentrations of CL‐4S amphiphile (0.005 mM to 1 mM) c) ^31^P‐NMR spectra of samples. d) Morphology of self‐assembled bilayer structures from MD simulations at different CL‐4S concentrations after 4.5 µs.

At first, as shown in Figure S15, 4S‐Nanodiscs displayed a temperature‐dependent behavior. When temperature was greater than or equal to 50 °C, the increased mobility of the bilayer could lead to nanodisc structural disability and result in lamellar phase.^[^ ^34^ ^]^ Then, the concentration dependence of morphology transitions in DMPC/CL/CL‐4S system was investigated via experimental analyses and MD simulations. As shown in Figure 5a,b, Cryo‐TEM and DLS results show the maintenance of stable vesicles at CL‐4S amphiphilic concentrations of 0.005 – 0.05 mM. However, upon increasing CL‐4S concentration to 0.25 mM, DLS revealed a sharp decrease in particle size, marking the onset of nanodiscs formation, which remained stable up to 1 mM concentration.^[^ ^35^ ^]^ To support these findings, we used ^31^P‐NMR spectroscopy, a technique highly responsive to changes in lipid aggregate size (Figure 5c). At 0 mM CL‐4S, no detectable signal was observed, suggesting large vesicles with slow rotational diffusion.^[^ ^35^ ^]^ By contrast, a distinct isotropic peak appeared at 0.5 mM and intensified at higher concentrations, pointing to the presence of nanodiscs, which tumble more rapidly in solution. Figure 5d shows the self‐assembled structures obtained in MD simulations with different ratios of CL‐4S amphiphile compared with DMPC and CL. The morphology transition is captured by increasing the CL‐4S content in the simulation box, mirroring the experimental results.

The simulation results demonstrate that the vesicle formation mechanism involves the merging of small bilayers, followed by the bending and closure of large bilayers into spherical structures (Figures S18–S23 and Tables S1–S4, in which Figure S23 highlights this mechanism). In this process the flexibility of the bilayer is crucial; in fact, less rigid systems tend to form smaller vesicles due to their lower bending energy. In contrast, increased bilayer rigidity, obtained for systems with higher concentrations of cholesterol (Figure S20) or CL‐1S (Figure S22), leads to the formation of larger vesicles.

In the case of CL‐4S, simulation and experimental observations agree that at low concentrations, the amphiphile cannot sufficiently stabilize the hydrophobic rim, thus preventing nanodiscs stabilization, similarly to CL‐1S. However, as the concentration of CL‐4S increases, the excess negative charge localizes at the disc rim, stabilizing the edge and promoting nanodiscs formation. Additionally, the electrostatic repulsion between these highly charged rims prevents nanodiscs–nanodiscs fusion, thereby suppressing the formation of larger bilayer structures that would otherwise bend and close into vesicles.

### Virus Binding and Inhibition

Supramolecular self‐assembly shows promising potential as dynamic virus inhibitor for the ability to adapt to different virus morphology and receptors.^[^ ^36^ ^]^ Considering sulfate groups’ important role in binding to herpes simplex virus‐1 (HSV‐1),^[^ ^37^, ^38^, ^39^, ^40^ ^]^ next we evaluated the potential of sulfated vesicles and nanodiscs being HSV‐1 inhibitors. First, we evaluated the compatibility of sulfated nanostructures toward Vero Cells, which is the cell line used in HSV‐1 infection assays in this study. In Figure S16, at 4 mM concentration of CL‐1S and 1S‐Vesicles, Vero cells enjoyed ∼100% cellular viability. Additionally, we found that CL‐4S surfactant itself at 1 mM was ∼ 100% toxic against Vero cells, which may be explained by the strong interactions between the highly‐charged amphiphiles and cellular membrane.^[^ ^41^ ^]^ Intriguingly, in 4S‐Nanodiscs with 1 mM CL‐4S, ∼100% cellular compatibility was observed, suggesting that the toxicity issue could be addressed by encasing CL‐4S in DMPC/CL lipid bilayer structures.

**Figure 6:**
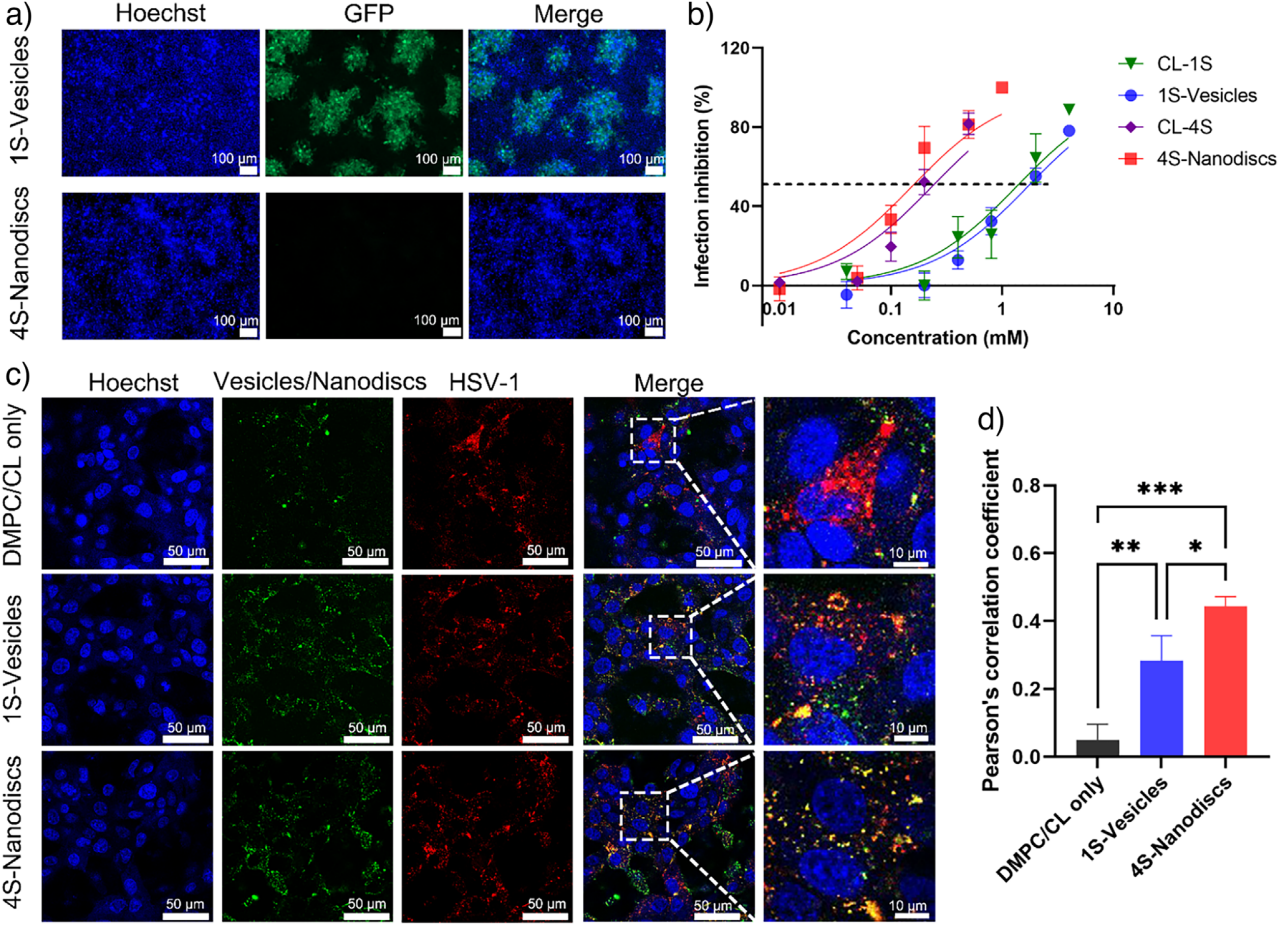
a) Representative fluorescent images for the HSV‐1‐infected Vero cells with 1S‐Vesicles (DMPC/CL‐1S/CL = 4/2/0.67 mM) and 4S‐Nanodiscs (DMPC/CL‐4S/CL = 4/0.5/0.67 mM). Scale bar=100 µm. b) Infection inhibition of HSV‐1 by different systems at different concentrations (MOI 0.1). Values are presented as mean ± SD, *n* = 3. c) Fluorescent images showing colocalization of HSV‐1 with vesicles/nanodiscs. Vero cells were incubated with R18‐labelled HSV‐1 (red), which was pre‐mixed with DiO‐labelled vesicles/nanodiscs (green) at 37 °C. Cell nuclei were stained with Hoechst (blue). Yellow spots represent colocalization between virus and vesicles/nanodiscs and are indicated by white square. d) Calculation of the pearson coefficient of colocalization for different samples. Values are expressed as mean ± SD, *n* = 3. Asterisks indicate statistical significance determined by two‐way ANOVA (*p < 0.05, **p < 0.01, and ***p < 0.001). For the abbreviations: R18: Octadecyl Rhodamine B Chloride; DiO: 3,3′‐Dioctadecyloxacarbocyanine Perchlorate.

The antiviral ability was carried out using a green fluorescent protein (GFP) modified HSV‐1. Infected Vero cells were detected by fluorescent microscopy. In Figure 6a,b and Figure S17, the DMPC/CL did not inhibit HSV‐1 infection, suggesting that the inhibition ability in 1S‐Vesicles and 4S‐Nanodiscs systems derived from sulfate groups. Additionally, we found that at the same amount of sulfate groups, the 4S‐Nanodiscs showed lower half maximal inhibitory concentration (IC_50_) values.

Given to previous studies,^[^ ^42^ ^]^ we hypothesize that the nanodiscs binds stronger than vesicle due to its smaller size. For a proof, we performed a co‐localization assay. Herein, the HSV‐1 and nanodiscs/vesicles were labelled by Octadecyl Rhodamine B Chloride (R18) and 3,3′‐Dioctadecyloxacarbocyanine Perchlorate (DiO), respectively. After co‐incubation, they were incubated with Vero cells, and then imaged via fluorescent microscope. In Figure 6c,d, rare orange colocalization indicated by overlapping spots was found in DMPC/CL only, while significant colocalization signals could be noticed and were highlighted by white square in 1S‐Vesicles and 4S‐Nanodiscs. Meanwhile, the 4S‐Nanodiscs binds stronger to virus particle than 1S‐Vesicles, which is one reason for its better virus inhibitory activities than the 1S‐Vesicles.

## Conclusion

The structural design and modular synthetic approach described here enabled the synthesis of cholesterol‐based surfactants (CL‐4S and CL‐4OH) that can be readily incorporated into membrane‐mimetic environments constructed of DMPC/CL lipid bilayers. Further analyses using DLS, cryo‐TEM, and MD simulations revealed that nanodiscs formation depends on the number of charges on surfactants, where the charges were introduced through post‐functionalization of oligo‐glycerol units. Mean‐field theoretical analysis revealed that multivalent charges preferentially localize at the edges of the nanodiscs. Moreover, MD simulations suggest that this charge distribution plays a crucial role in governing the supramolecular behavior of the system. Additionally, the resulting nanodiscs systems exhibited antiviral activity against HSV‐1, slightly outperforming other sulfated nanostructures, possibly in a size‐dependent manner.

By introducing site‐specific chemical modifications to surfactants and integrating them into lipid bilayers, we can engineer lipid‐based materials that adopt vesicular or self‐assembled nanodisc morphologies. This approach to designing tailored, surfactant‐driven supramolecular biomaterials offers a versatile platform for creating new classes of lipid materials and addressing diverse biomedical challenges, for example, drug delivery and infectious disease treatment. Furthermore, by systematically dissecting the compositional parameters that dictate nanodisc formation, our work provides fundamental insights into how supramolecular assemblies can be rationally programmed through precise compositional control.

## Supporting Information

Supporting Information is available free of charge: materials and methods for experiments and simulations, NMR figures, cytotoxicity analysis, related biological experimental protocols, and simulation protocols.

## Conflict of Interests

The authors declare no conflict of interest.

## Supporting information



Supporting Information

Supporting Information

## Data Availability

The data that support the findings of this study are available in the Supporting Information of this article.
