# Supplementary material for: Charge‐Driven Self‐Assembly of Cholesterol Surfactants into Biofunctional Nanodiscs with Antiviral Activity
Source: Angew Chem Int Ed Engl. 2025 Nov 13;65(1):e16207. doi: 10.1002/anie.202516207 (PMC12759236; doi:10.1002/anie.202516207)
Supplement: Supplementary file 1 — Supporting Information [file ANIE-65-e16207-s002.docx]

**Supporting information**

**Charge-Driven** **Self-Assembly of Cholesterol Surfactants into Biofunctional Nanodiscs with Antiviral Activity**

Yanping Long,^[a]^ Seyyed Mohammad Mousavifard,^[d]^ Xianfeng He,^[e]^ Roland R. Netz,^[e]^ Hesam Makki,*^[c]^ Mathias Dimde,^[a,b]^ Chuanxiong Nie,*^[a]^ Abhishek K. Singh,*^[a]^ Rainer Haag*^[a]^

**Table of Contents**

[1. Experimental Methods 2](#_Toc204189454)

[1.1 Materials & Methods 2](#_Toc204189455)

[1.2 Chemical synthesis and characterization 3](#_Toc204189456)

[1.3 Critical micelle concentration (CMC) 6](#_Toc204189457)

[1.4 Supramolecular formulations 6](#_Toc204189458)

[1.5 Biological studies 6](#_Toc204189459)

[2. Experimental results 8](#_Toc204189460)

[3. Simulation Method and Results 20](#_Toc204189461)

[3.1. Molecular dynamics (MD) simulations 20](#_Toc204189462)

[3.1.1. Coarse-Grained Force Field Parameterization 21](#_Toc204189463)

[3.1.2. Validation of CG Parameters 25](#_Toc204189464)

[3.2. 2D charge distribution on nanodiscs 27](#_Toc204189465)

[3.2.1 System Hamiltonian 28](#_Toc204189466)

[3.2.2 Mean-Field Methodology 28](#_Toc204189467)

[3.2.3 Monte Carlo Methodology 28](#_Toc204189468)

[3.2.4 Monte Carlo Snapshots 29](#_Toc204189469)

[3.2.5 Effect of Screening Parameter κ 29](#_Toc204189470)

[4. Supporting Videos 31](#_Toc204189471)

# 1. Experimental Methods

## 1.1 Materials & Methods

***Materials:***

Cholesterol, Sodium hydride (NaH), Triethylamine (TEA), methane sulphonyl chloride (MsCl), allyl bromide, Cupper (I) acetate, sodium-3-mercapto-1-propanesulfonate (MPS), 2,2-Dimethoxy-2-phenylacetophenone (DMPA), 1,2-Dimyristoyl-sn-glycero-3-phosphocholine (DMPC), cholesterol sulfate, Nile Red, Octadecyl Rhodamine B Chloride (R18), and 3,3´-dioctadecyloxacarbocyanine, perchlorate (DiO) were purchased from Merck KGaA, Darmstadt, Germany and/or its affiliates and used without any further purification, unless otherwise stated. Dowex 50WX8 200-400 H was also purchased from Merck KGaA, Darmstadt, Germany, prior to its usage, washing with Methanol was required. Propargyl bromide was purchased from TCI Deutschland GmbH. Sodium azide was also procured from Fischer Scientific. The solvents used herein, i.e., diethyl ether (100%), N, N-dimethylformamide (99.8%), DCM (99%) and ethyl acetate were bought from Merck KGaA, Darmstadt, Germany. The extra dry tetrahydrofuran (THF) and extra dry DMF were both obtained from Fischer Scientific. UV bypass filter with 365 nm monochrome light and 49 mm diameter was purchased from Vision Light Tech B.V. The protected G1 was provided by AG Haag group.

***Instruments:***

NMR: The Jeol Eclipse 500 MHz (Tokyo, Japan) or a Bruker AVANCE III 700 MHz spectrometer (Billerica,MA, USA) instruments were used to measure all the NMR spectra of all the compounds (1H and 13C) reported here were recorded at 400 K. Chemical shifts 𝛿 were reported in ppm and the deuterated solvent peak was used as a standard. DLS and Zeta potential: Multi Angel Light Scattering (MALS, Malvern Panalytical, Germany) Instruments was used to measure the DLS and Zeta potential of all relevant compounds reported in this work. Cryo-TEM: Perforated carbon film-covered microscopical 200 mesh grids (R1.2/1.3 batch of Quantifoil, MicroTools GmbH, Jena, Germany) were cleaned with chloroform and hydrophilized by 60 s glow discharging at 10 mA in a Safematic CCU-010 device (safematic GmbH, Zizers, Switzerland). Subsequently, 4 μl aliquots of the sample solution were applied to the grids. The samples were vitrified by automatic blotting and plunge freezing with a FEI Vitrobot Mark IV (Thermo Fisher Scientific Inc., Waltham, Massachusetts, USA) set to 20°C and 100% humidity using liquid ethane as cryogen. The vitrified specimens were transferred to the autoloader of a FEI TALOS ARCTICA electron microscope (Thermo Fisher Scientific Inc., Waltham, Massachusetts, USA). This microscope is equipped with a high-brightness field-emission gun (XFEG) operated at an acceleration voltage of 200 kV. Micrographs were acquired on a FEI Falcon 3 direct electron detector (Thermo Fisher Scientific Inc., Waltham, Massachusetts, USA) using a 100 μm objective aperture.

## 1.2 Chemical synthesis and characterization

***Synthesis of protected G1-azide (*pG1-N_3_)*:***

Protected G1 (pG1) was characterized by NMR. Started from pG1, the pG1-N_3_ was synthesized via mesyllation and azidation. For the mesyllation, dried pG1 (10 g, 0.031 mol) was dissolved in DCM (100 mL) followed by addition of TEA (8.7 mL, 0.54 mol, 2.0 eq. with respect to OH group) and cooled to 0 °C. Then methane sulphonyl chloride (MsCl) (3.6 mL, 0.047 mol, 1.3 eq. with respect to OH group) was added dropwise using a syringe at 0 °C and kept stirred for 2 h. Then, the reaction was stopped, and we purified and concentrated the protected G1-OMs (pG1-OMs) (11.95 g, 96%) via extraction and rotary evaporation. For the azidation, pG1-OMs (11.95 g, 0.03 mol) was dissolved in dry DMF (80 mL) in a 250 mL flask equipped with a refluxing condenser. NaN_3_ (3.9 g, 0.06 mol, 2.0 eq. with respect to OMs group) was added to the reaction mixture and stirred at 80 °C for 48 h. The solvent DMF from the reaction mixture was evaporated using a rotary evaporator, and the pG1-N_3_ was purified via extraction and column chromatography (5.9 g, 57%).

**pG1:**

**^1^H NMR** (500 MHz, CD_3_OD) δ 4.32 – 4.24 (m, 2H), 4.09 - 4.06 (m, 2H), 3.88 (s, 1H), 3.77 - 3.74 (m, 2H), 3.60 – 3.49 (m, 8H), 1.41 (s, 6H), 1.35 (s, 6H).

**^13^C NMR** (126 MHz, CD_3_OD) δ 110.45, 76.16, 76.05, 73.96, 73.90, 73.45, 73.40, 73.35, 70.61, 70.48, 67.68, 67.53, 67.41, 27.11, 27.01, 25.69, 25.61 ppm.

**pG1-N_3_:**

**^1^H NMR** (500 MHz, CD_3_OD) δ 4.29 – 4.22 (m, 2H), 4.09 – 4.02 (m, 2H), 3.77 - 3.72 (m, 3H), 3.69 – 3.50 (m, 8H), 1.39 (s, 6H), 1.34 (s, 6H).

**^13^C NMR** (126 MHz, CD_3_OD) δ 110.51, 76.11, 76.01, 73.19, 73.13, 72.18, 72.07, 67.58, 67.44, 67.32, 61.96, 27.08, 26.99, 25.70, 25.62 ppm.

***Synthesis of G1-N_3_-4 allyl:***

The synthesis of G1-N_3_-4 allyl was proceeded with deprotection and allylation. For the deprotection: the pG1-N_3_ (5.9 g, 0.017 mol) was dissolved in MeOH (30 mL) in 100 mL flask, then 20 wt% (1.18 g) of washed Dowex 50WX8 200-400 (H) was added to the flask at 50 °C and kept stirred overnight. The product G1-N_3_ (4.5 g, 99%) was collected via filtration and rotary evaporation. For the allylation, in a 250 mL round bottom flask, sodium hydride (NaH) (4.16 g, 10 equiv.) was dissolved in 100 mL DMF in argon atmosphere. Then, G1-N_3_ (4.51 g, 0.017 mol, 1.0 equiv.) was also dissolved in 50 mL DMF, transferred to NaH solution dropwise, magnetically stirred for 10 min on ice condition and 30 min at 50 ^o^C. Finally, allyl bromide (12.9 ml, 8.0 equiv.) was added via syringe, and kept stirring overnight at 50 ^o^C for 24 h under argon atmosphere. Then, the product G1-N_3_-4 allyl was purified and concentrated via extraction, column chromatography and rotary evaporation (41%, 3 g).

**G1-N_3_:**

**^1^H NMR** (600 MHz, CD_3_OD) δ 3.85 – 3.79 (m, 3H), 3.74 – 3.53 (m, 12H) ppm.

**^13^C NMR** (151 MHz, CD_3_OD) δ 164.93, 79.69, 79.62, 73.79, 73.65, 73.61, 72.68, 72.23, 72.08, 72.04, 71.93, 71.81, 64.27, 64.25, 61.91, 52.70, 37.06, 31.75 ppm.

**G1-N_3_-4 allyl:**

**^1^H NMR** (400 MHz, CD_3_OD) δ 6.03 – 5.91 (m, 4H), 5.38 – 5.30 (m, 4H), 5.24 – 5.18 (m, 4H), 4.21 – 4.18 (m, 4H), 4.07 – 4.05 (m, 4H), 3.75 – 3.56 (m, 15H) ppm.

**^13^C NMR** (101 MHz, CD_3_OD) δ 136.43, 117.17, 116.97, 78.44, 73.24, 72.38, 72.23, 72.06, 70.95, 61.97 ppm.

**MS (ESI)** m/z = 448.2, 464.2; [M+Na]^+^ (calcd. for C_21_H_35_N_3_NaO_6_: 448.53). [M+K]^+^ (calcd. For C_21_H_35_N_3_KO_6_: 464.53).

***Synthesis of CL-alkyne:***

Cholesterol (CL) was characterized by ^1^H NMR. For the synthesis, in a 250 mL round bottom flask, sodium hydride (NaH) (0.37 g, 2.0 equiv.) was dissolved in 20 mL tetrahydrofuran (THF) in argon atmosphere. Then, CL (3 g. 1.0 equiv.) was also dissolved in 20 mL THF, transferred to NaH solution dropwise, magnetically stirred for 10 min on ice condition and 30 min at 50 ^o^C. Finally, propargyl bromide (1 ml, 1.5 equiv.) was added via syringe, and kept stirring overnight at 50 ^o^C for 48 h under argon atmosphere to obtain CL-alkyne. The other day, stop the reaction, purify and concentrate the CL-alkyne via extraction, rotary evaporation and column chromatography. (1.75 g, 53%).

**CL:**

**^1^H NMR** (500 MHz, CD_2_Cl_2_) δ 5.34 (s, 1H), 3.45 (s, 1H), 2.26 – 2.17 (m, 2H), 2.06 – 1.92 (m, 2H), 1.88 – 1.74 (m, 3H), 1.59 – 0.86 (m, 34H), 0.68 (s, 3H) ppm.

**^13^C NMR** (126 MHz, CD_2_Cl_2_) δ 141.37, 121.85, 72.05, 57.19, 56.56, 50.60, 42.74, 42.68, 40.21, 39.88, 37.67, 36.87, 36.58, 36.21, 32.31, 32.29, 32.11, 28.60, 28.42, 24.64, 24.18, 22.95, 22.70, 21.47, 19.58, 18.89, 12.00 ppm.

**CL-alkyne:**

**^1^H NMR** (500 MHz, CD_2_Cl_2_) δ 5.36 – 5.35 (m, 1H), 4.16 (s, 2H), 3.36 – 3.30 (m, 1H), 2.42 – 2.41 (m, 1H), 2.34 - 2.15 (m, 2H), 2.04 – 1.95 (m, 3H), 1.89 – 1.82 (m, 3H), 1.59 – 1.42 (m, 9H), 1.36 – 1.33 (m, 3H), 1.17 – 1.06 (m, 7H), 1.01 – 0.99 (m, 4H), 0.92 - 0.91 (m, 2H), 0.87 – 0.85 (m, 7H), 0.68 (s, 3H) ppm.

**^13^C NMR** (126 MHz, CD_2_Cl_2_) δ 141.06, 122.15, 81.07, 78.57, 73.70, 57.21, 56.59, 55.34, 50.63, 42.70, 40.22, 39.90, 39.16, 37.54, 37.20, 36.60, 36.23, 32.36, 32.29, 28.62, 28.50, 28.43, 24.66, 24.22, 22.98, 22.73, 21.47, 19.56, 18.92, 12.03 ppm.

***Synthesis of CL-4 allyl:***

G1-N_3_-4 allyl (1 g, 0.00235 mol, 1.0 equiv.) and CL-alkyne (1.2 g, 0.0028 mol, 1.2 equiv. per azide) was dissolved in 15 mL THF. To this solution, Cupper (I) acetate (0.085 g, 0.2 equiv. per azide) was added and stirred well. The overall reaction mixture was degassed with argon for 30 min and allowed to stir at 50 ^o^C for 24 h under argon atmosphere. After completion of reaction as monitored by TLC, the reaction mixture was concentrated under reduced pressure. Then extraction, rotary evaporation and column chromatography were applied to purify and concentrate CL-4 allyl (1.47 g, 73%).

**CL-4 allyl:**

**^1^H NMR** (500 MHz, CD_3_COCD_3_) δ 7.95 (m, 1H), 5.92- 5.84 (m, 4H), 5.36 – 5.34 (m, 1H), 5.28 – 5.22 (m, 4H), 5.13 – 5.07 (m, 4H), 4.99 – 4.94 (m, 1H), 4.61 - 4.57 (m, 2H), 4.09 – 4.05 (m, 4H), 3.98 – 3.92 (m, 7H), 3.62 – 3.58 (m, 3H), 3.56 – 3.53 (m, 2H), 3.44 – 3.42 (m, 3H), 3.32 – 3.25 (m, 1H), 2.86 – 2.80 (m, 2H), 2.41 – 2.13 (m, 2H), 1.99 – 1.84 (m, 4H), 1.67 – 1.34 (m, 12H), 1.33 – 1.25 (m, 3H), 1.19 – 1.06 (m, 7H), 1.03 – 1.00 (m, 4H), 0.95 – 0.94 (m, 2H), 0.88 – 0.83 (m, 7H), 0.73 - 0.69 (m, 3H) ppm.

**^13^C NMR** (101 MHz, CD_3_COCD_3_) δ 145.92, 141.75, 136.77, 136.27, 136.21, 123.46, 122.18, 122.17, 116.40, 116.15, 79.10, 78.01, 72.64, 72.24, 71.58, 71.54, 71.15, 70.83, 61.37, 57.67, 57.08, 51.20, 43.12, 40.69, 40.27, 39.95, 38.06, 37.59, 36.99, 36.62, 32.77, 32.66, 24.96, 24.55, 22.87, 21.81, 19.78, 19.18, 12.27 ppm.

***Synthesis of* CL-4S:**

CL-4 allyl (1.3 g, 0.0015 mol, 1.0 equiv.), sodium 3-mercapto-1-propanesulfonate (2.14 g, 0.012 mol, 8.0 equiv.), 2,2-Dimethoxy-2-phenylacetophenone (0.154 g, 0.0006 mol, 0.4 equiv.) were mixed in Methanol/DMF mixture solvents and stirred well. The overall reaction mixture was degassed with argon for 10 min and allowed to stir at r.t. for 24 h under argon atmosphere and under UV illumination using an LED source at a fixed wavelength of 365 nm. After completion of reaction, the reaction mixture was concentrated under reduced pressure. Then, the reaction mixtures were purified by dialysis against
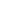
deionized water for 2 days using a 1 kDa *M*_w_ cutoff membrane. The water was removed using a rotary vacuum evaporator finally and freeze-drying. Eventually, the product (1.1 g) was obtained with a yield of (49%).

**CL-4S:**

**MS (ESI)** m/z = 1538.5; [M+3Na]^-^ (calcd. for C_64_H_111_N_2_Na_3_O_19_S_8_^-^: 1538.5599).

***Synthesis of CL-4OH:***

G1-N_3_ (0.5 g, 1.89 mmol, 1.1 equiv.) and CL-alkyne (0.73 g, 1.72 mmol, 1 equiv.) was dissolved in 20 mL THF. To this solution, Cupper (I) acetate (62.48 mg, 0.2 equiv.) was added and stirred well. The overall reaction mixture was degassed with argon for 30 min and allowed to stir at 50 ^o^C for 24 h under argon atmosphere. After completion of reaction as monitored by TLC, the reaction mixture was concentrated under reduced pressure. Then extraction, rotary evaporation and column chromatography were applied to purify and concentrate CL-4OH (0.75 g, 63%).

**CL-4OH:**

**MS (ESI)** m/z = 712.5, 728.5; [M+Na]^-^ (calcd. for C_40_H_67_N_2_NaO_7_: 712.4979), [M+K]^-^ (calcd. for C_40_H_67_N_2_KO_7_: 728.4979)

## 1.3 Critical micelle concentration (CMC)

CL-4S was dissolved in distilled water and 2-fold dilution was performed starting from 5 mg/mL. 10 µL of Nile red solution (1 mg/mL, in methanol) was added to 0.8 mL of surfactant solution in a 5-mL vial and rolled on a roller mixer overnight at room temperature under dark conditions. The fluorescence intensity of the solution was measured at 613 nm on a fluorescence spectrophotometer with excitation at 556 nm. The fluorescence intensity was plotted versus the logarithmic concentration of the surfactant solution.

## 1.4 Supramolecular formulations

***Fabrication of 4S-Nanodiscs:***

A mixed solution made of chloroform and methanol (8:2) was used to solubilize DMPC and CL as stock solution (DMPC: 100 mg/mL; CL: 100 mg/mL). CL-4S surfactant was solubilized in Tris/HCl buffer (10 mM Tris/HCl, 100 mM NaCl, 0.5 mM EDTA, 50 mM sodium cholate, pH 7.4) to stock concentration of 50 mg/mL. Then, certain volume of DMPC and CL stock solution were mixed, solvent was evaporated under with nitrogen gas. Then, we added CL-4S stock solution, and Tris/HCl buffer was supplemented to get solution composed of 8 mM DMPC/1 mM CL-4S/1.33 mM CL, and perform probe sonication for 3 min at 40 W.

***Fabrication of DMPC/CL only, 1S-Vesicles and 4OH-Vesicles:***

These fabrications are the same as 4S-Nanodiscs fabrication generally, except CL-1S is dissolved in DMSO.

***Characterizations:***

The vesicles and nanodiscs were characterized via DLS, Zeta potential and Cryo-TEM.

The samples concentration for measurements are 1S-Vesicles (DMPC/CL-1S/CL=8/4/1.33 mM), 4OH-Vesicles (DMPC/CL-4OH/CL=8/1/1.33 mM), and 4S-Nanodiscs (DMPC/CL-4S/CL=8/1/1.33 mM).

## 1.5 Biological studies

***Biosafety evaluation:***

Vero cells were pre-seeded in 96-well plate and incubated at 37 ^o^C and 5% CO_2_. At the next day, cells were incubated with surfactants, vesicles and nanodiscs of different concentrations for 24 h, afterwards, diluted CCK-8 solution (Sigma-Aldrich, Germany) was added to each well. After 2 h incubation, the mixture was detected by a microplate reader (Model 550, Bio-Rad) at 450 nm and analysed by a standard protocol to calculate the cellular toxicity as shown follows:

Cell viability (%) =$\left( \frac{\left| 450 \right|\left( compounds \right)-\left| 450 \right|\left( negativecontrol \right)}{\left| 450 \right|\left( positivecontrol \right)-\left| 450 \right|\left( negativecontrol \right)} \right)*100\text{\%}$

***HSV-1 binding assay:***

The HSV-1 inhibition performance of surfactants, vesicles and nanodiscs were investigated via pre-infection inhibition assay. Specifically, Vero cells were seeded into 96-well plates; after being washed with phosphate-buffered saline (PBS), the cells were incubated with samples of different concentrations for 45 min, followed by adding 10 µL of 10^6^ PFU/mL HSV-1. Finally, after 24 h incubation and fixation, Hoechst 33342 (Invitrogen) was used to stain cell nuclei and fluorescent images were acquired on a Zeiss Axio Observer Z1 microscope (ZEISS, Germany). The total cells and infected cells were counted via ImageJ, and the infection was then estimated by the ratio of infected cells in total cells.

The half maximal inhibitory concentration (IC_50_) was determined by plotting the infection ratio of Vero cells to the compound concentration. Curve fitting was done using GraphPad Prism 9 with [Agonist] vs. normalized response [Y=100*X/(EC50+X)].

***Colocalization of Vesicles/Nanodiscs and HSV-1 when incubated with Vero cells:***

Prior to staining, HSV-1 was purified by ultracentrifugation at 100,000 g for 2 h (4 ^o^C). For the colocalization experiment, purified HSV-1 and samples (DMPC/CL only, 1S-Vesicles, 4S-Nanodiscs) were labelled with Octadecyl Rhodamine B Chloride (R18), 3,3´-dioctadecyloxacarbocyanine perchlorate (DiO), respectively, and purified through spin column (PD SpinTrap^TM^ G-25 Columns, GE Healthcare) to remove extra dyes. Then, the labelled HSV-1 and samples were premixed for 45 min with continuous shaking, transferred to Vero cells for 2 h, and then fixed with 4% formaldehyde and stain with Hoechst 33342 (Invitrogen). The fluorescent images were are acquired on SP8 Lighting confocal microscope (Leica, Germany) with a 63× objective.

# 2. Experimental results


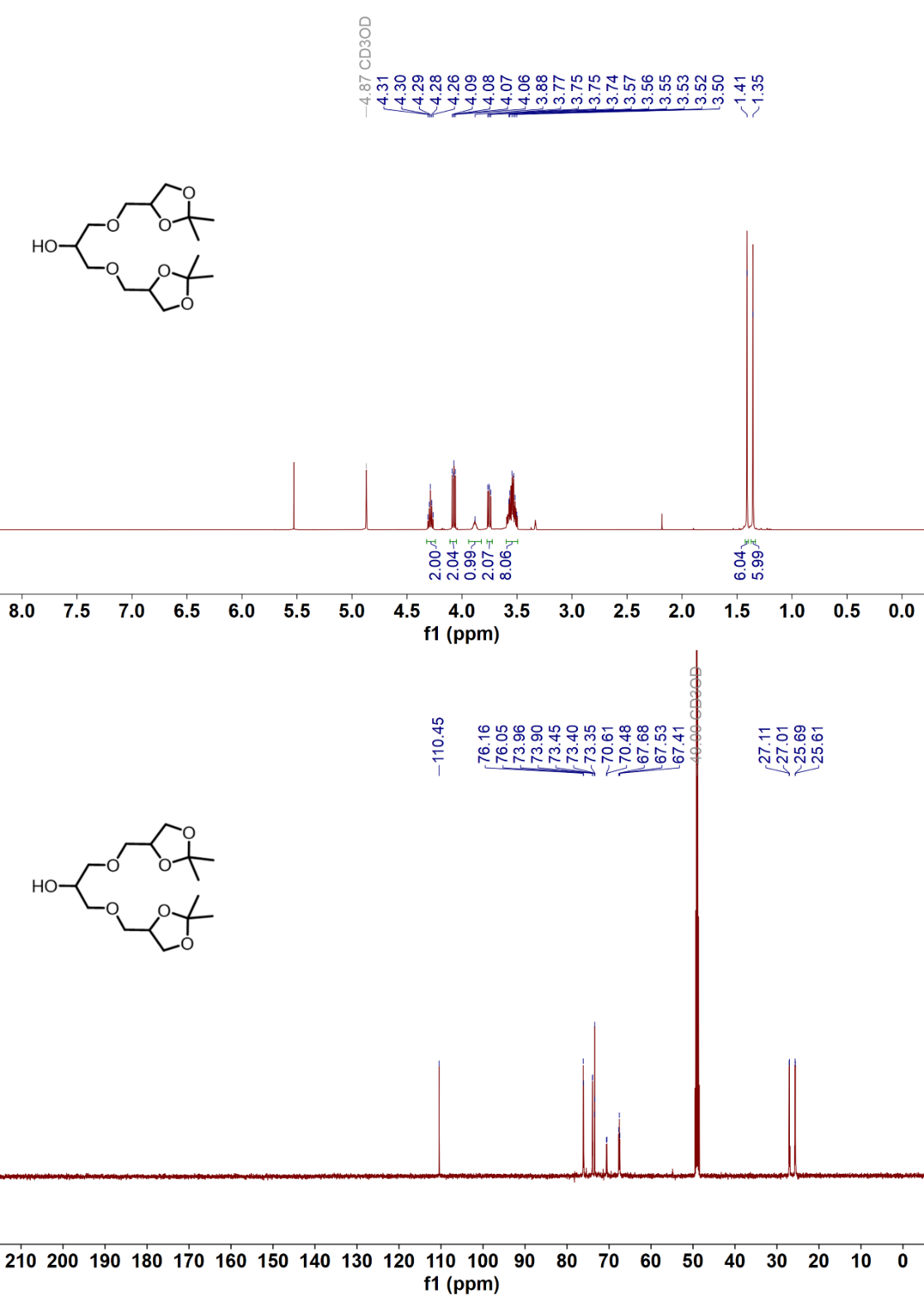


**Figure S1.** ^1^H and of ^13^C NMR of pG1 in CD_3_OD.


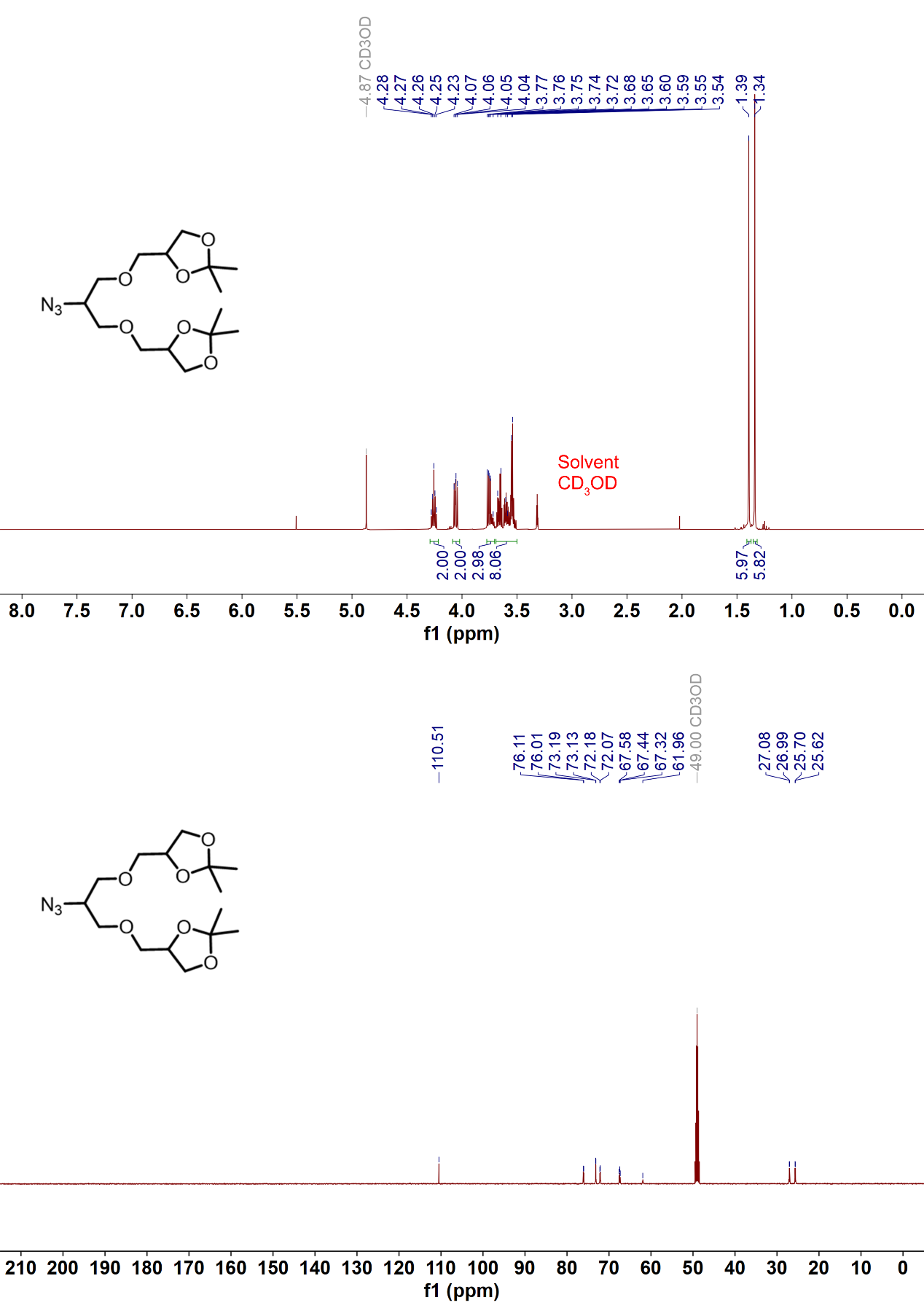


**Figure S2.** ^1^H and of ^13^C NMR of pG1-N_3_ in CD_3_OD.


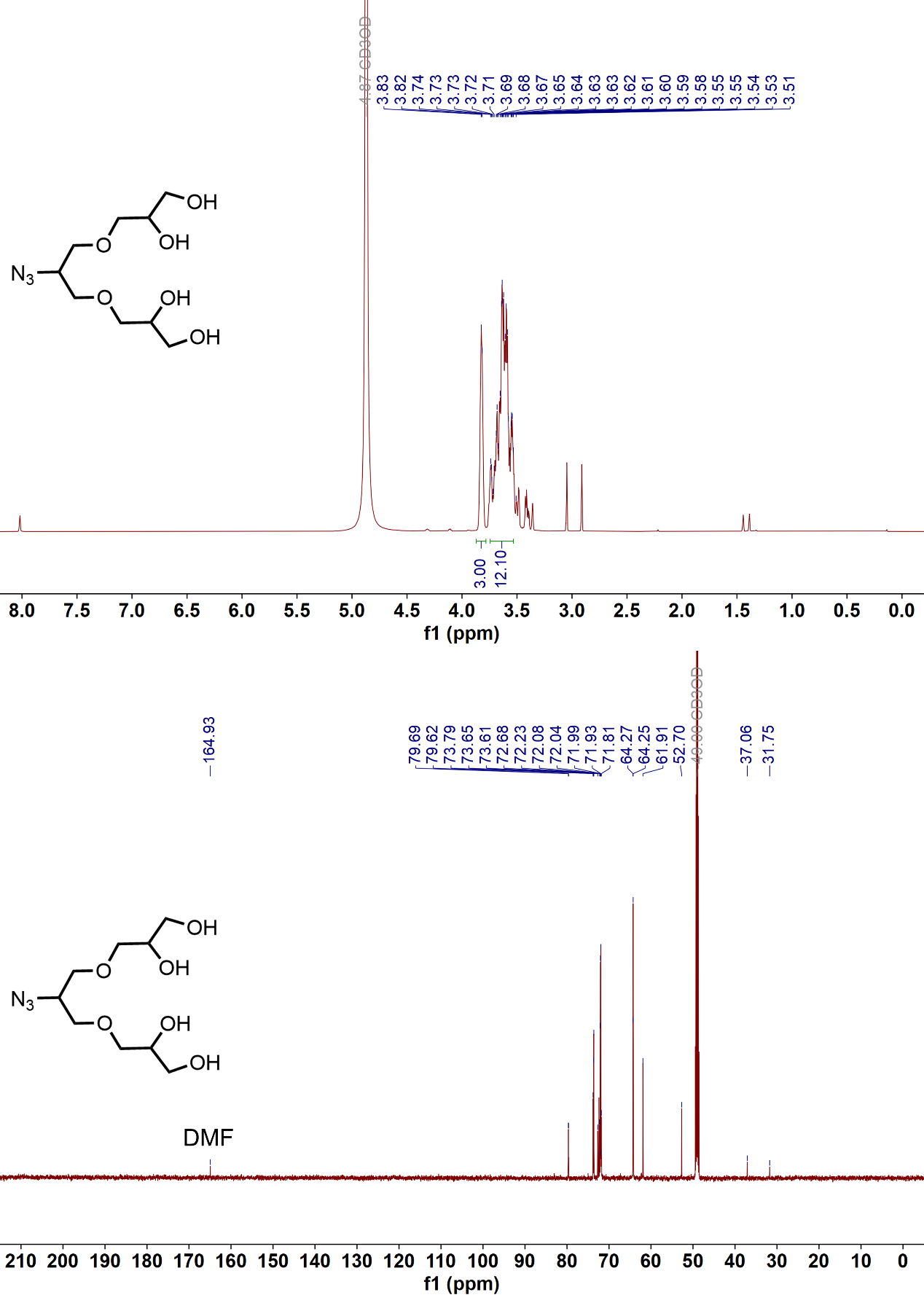


**Figure S3.** ^1^H and of ^13^C NMR of G1-N_3_ in CD_3_OD.


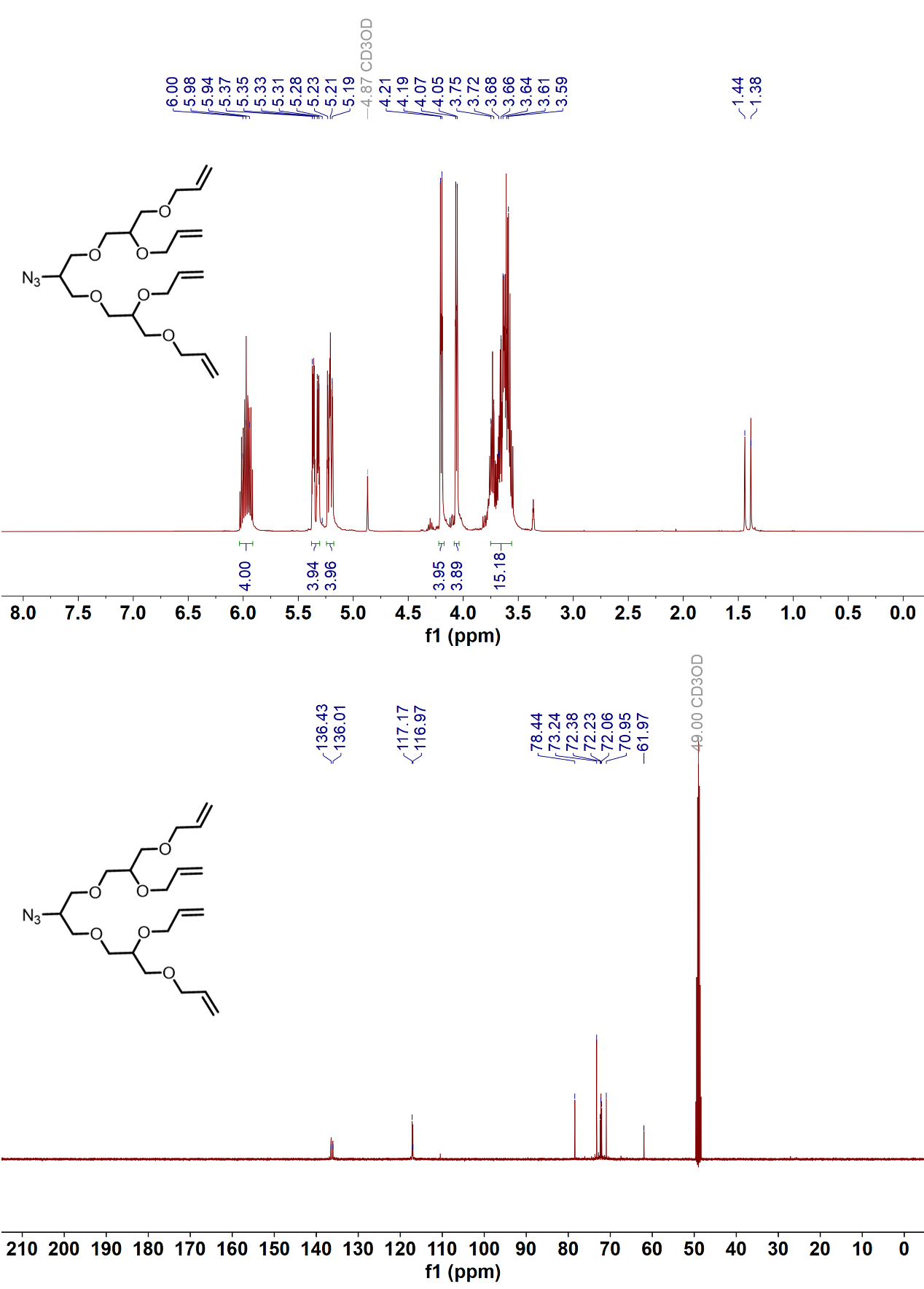


**Figure S4.** ^1^H and of ^13^C NMR of G1-N_3_-4 allyl in CD_3_OD.


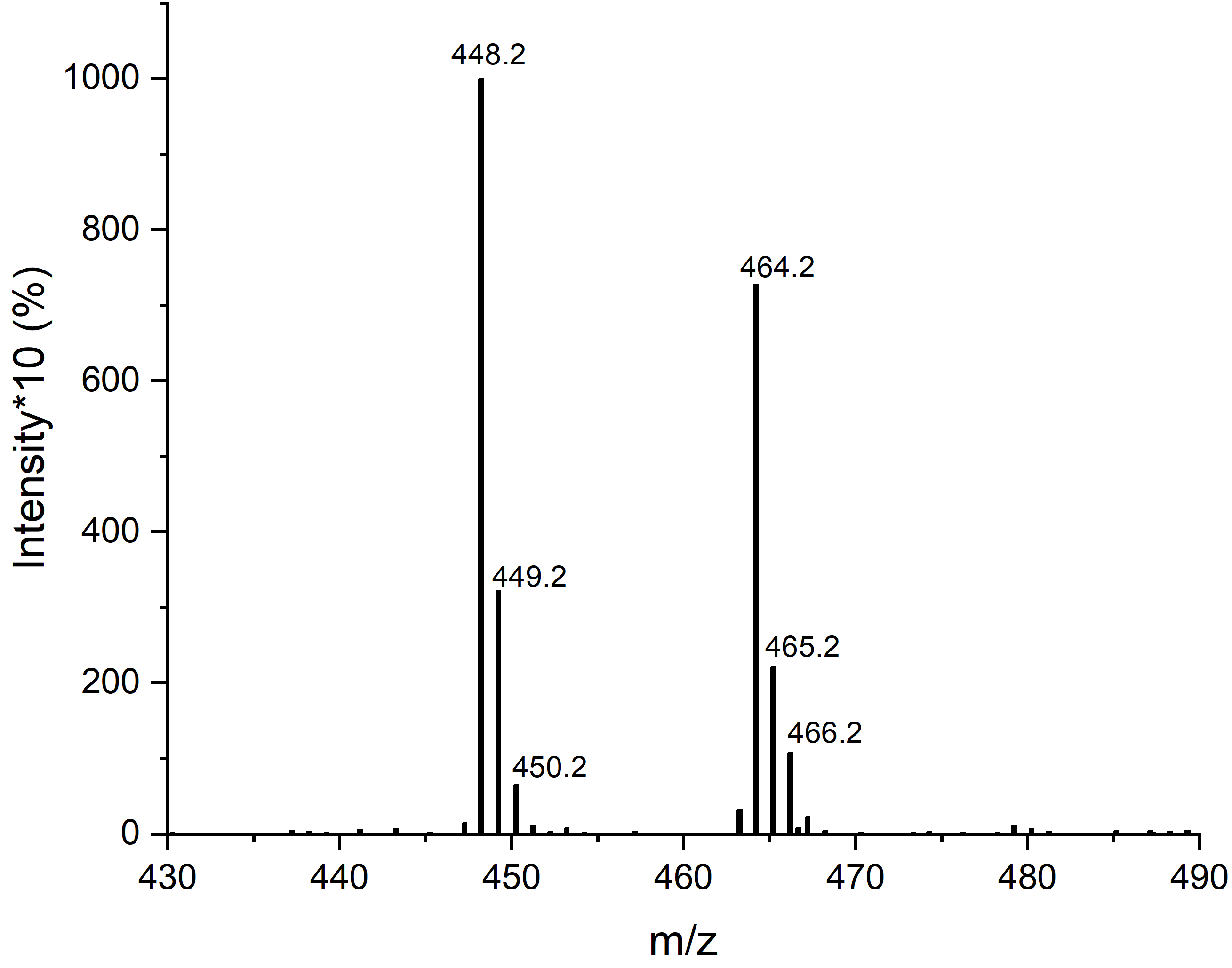


**Figure S5.** Mass spectra for G1-N_3_-4 allyl. [M+Na]^+^ (calcd. For C_21_H_35_N_3_NaO_6_: 448.53). [M+K]^+^ (calcd. For C_21_H_35_N_3_KO_6_: 464.53).


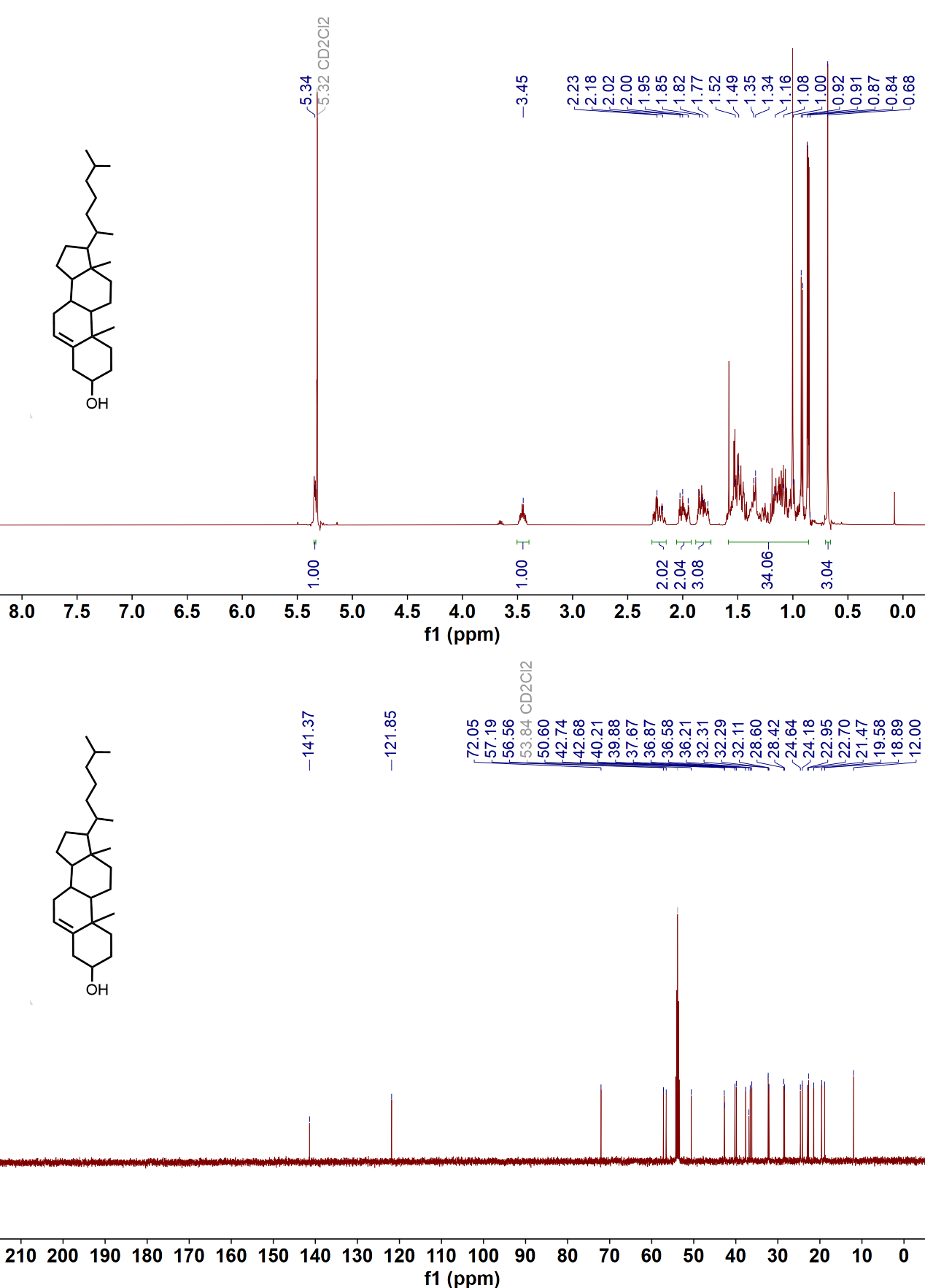


**Figure S6.** ^1^H and of ^13^C NMR of CL in CD_2_Cl_2_.


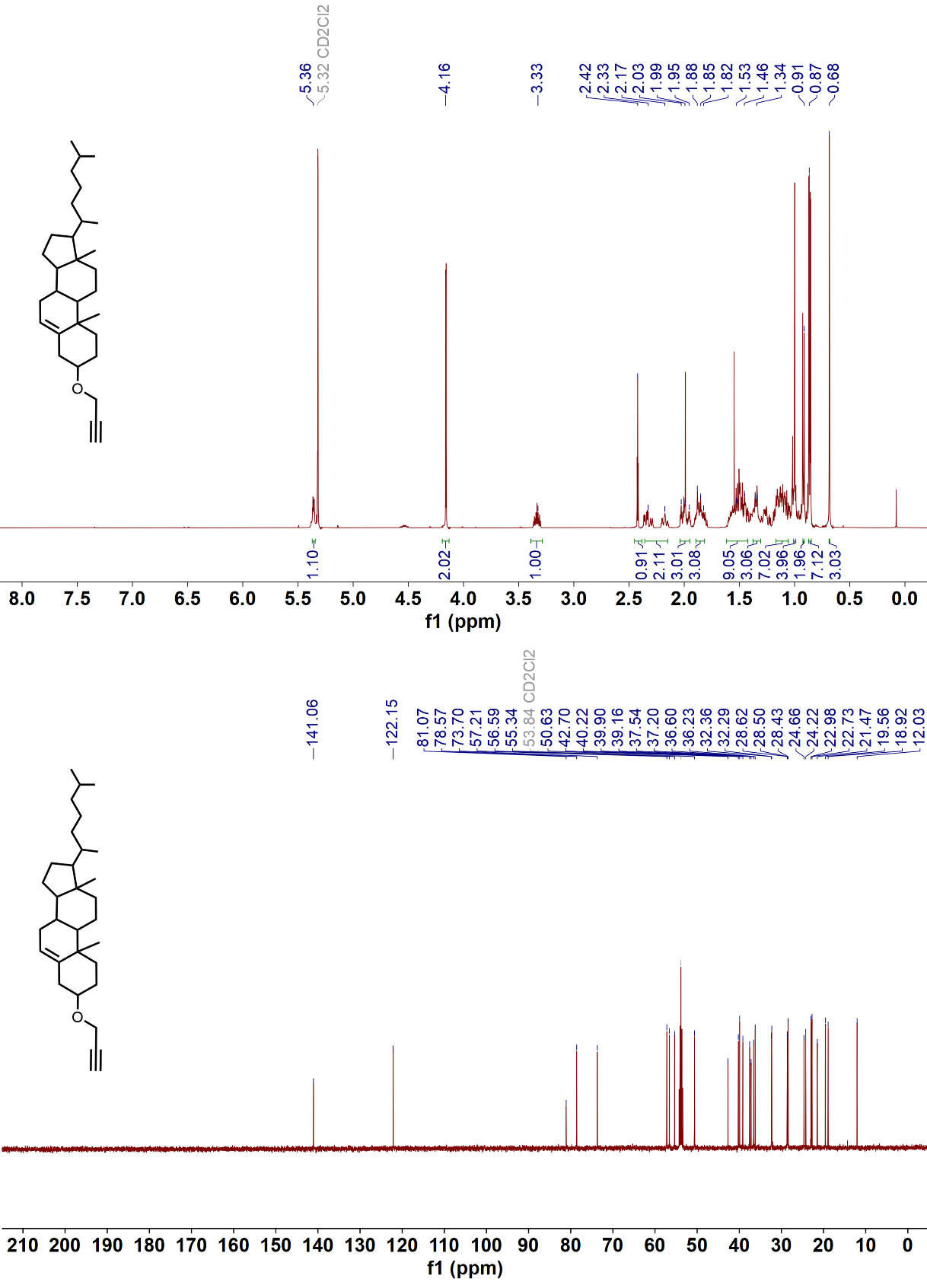


**Figure S7.** ^1^H and of ^13^C NMR of CL-alkyne in CD_2_Cl_2_.


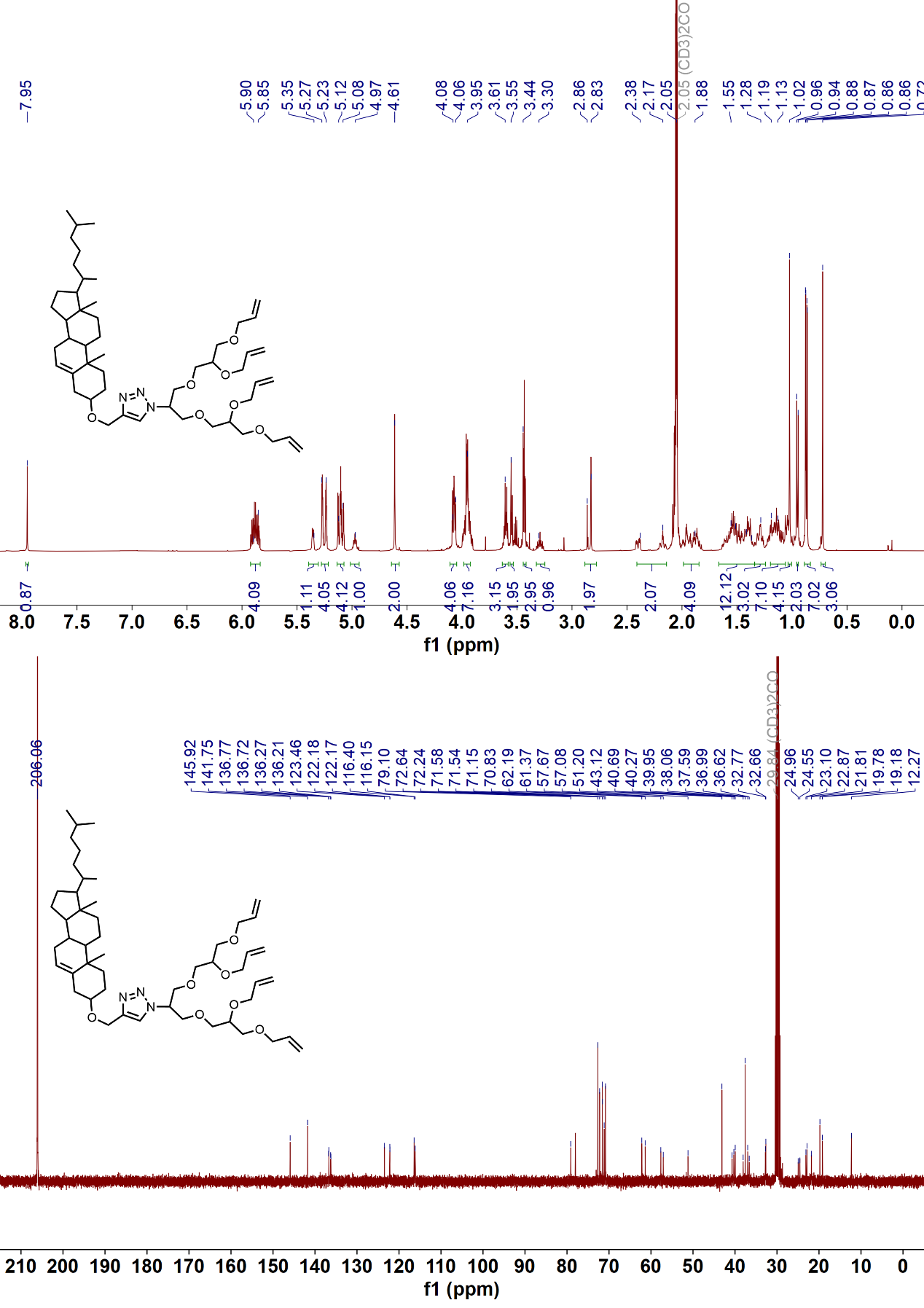


**Figure S8.** ^1^H NMR of CL-4 allyl in CD_3_COCD_3_.


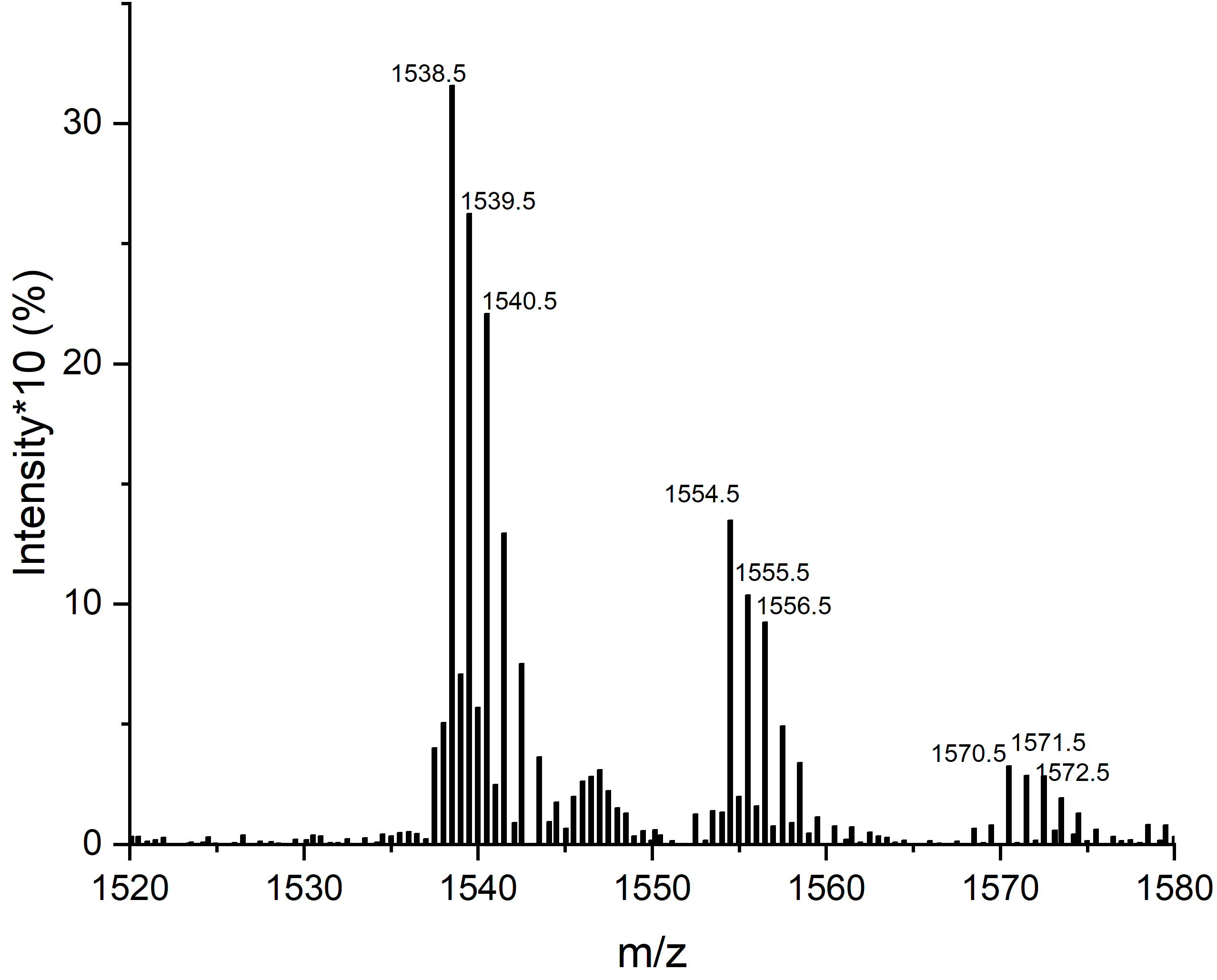


**Figure S9.** Mass spectra for CL-4S. [M+3Na]^-^ (calcd. for C_63_H_111_N_3_Na_3_O_19_S_8_^-^: 1538.5599).


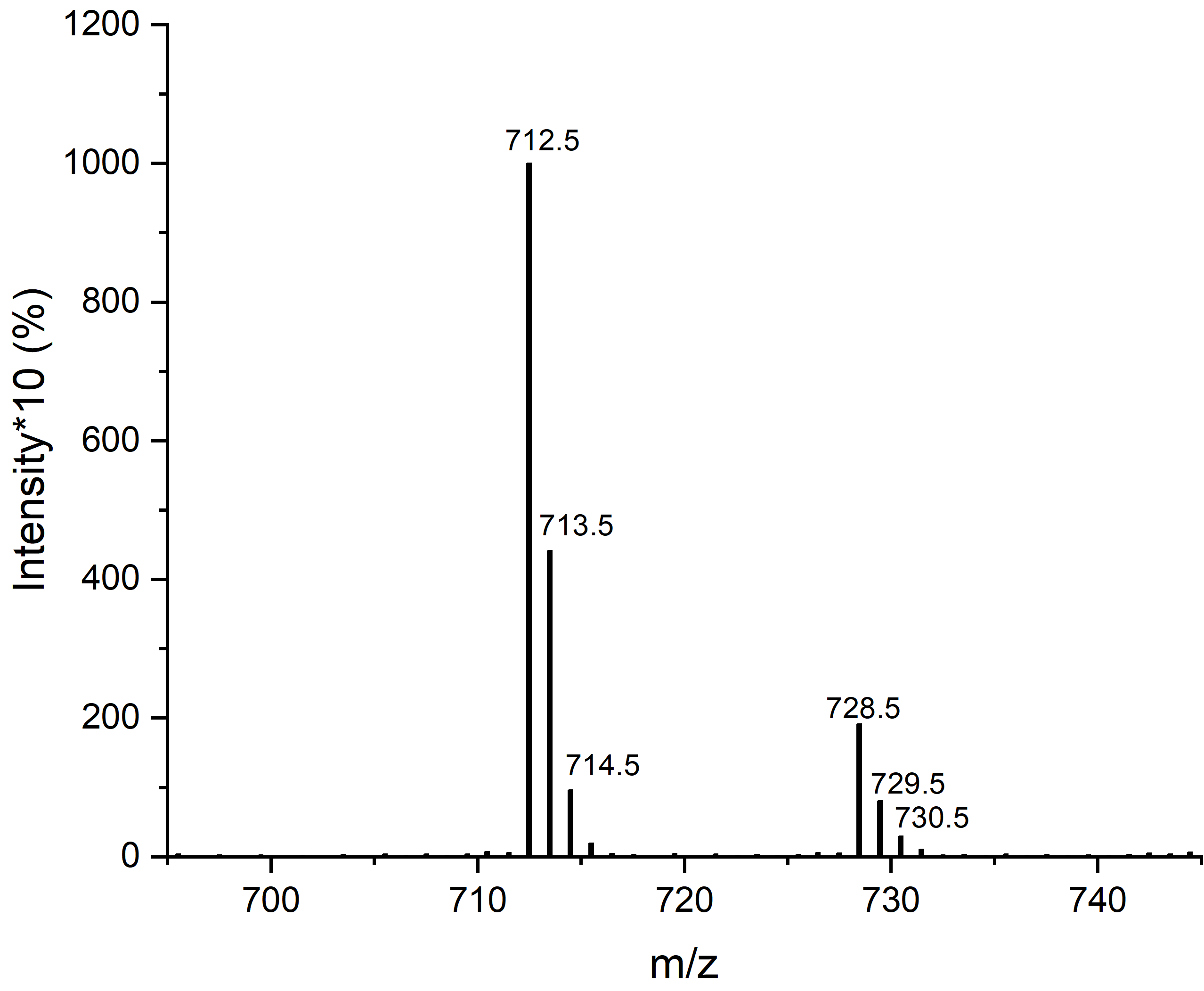


**Figure S10.** Mass spectra for CL-4OH. [M+Na]^-^ (calcd. for C_40_H_67_N_2_NaO_7_: 712.4979), [M+K]^-^ (calcd. for C_40_H_67_N_2_KO_7_: 728.4979).


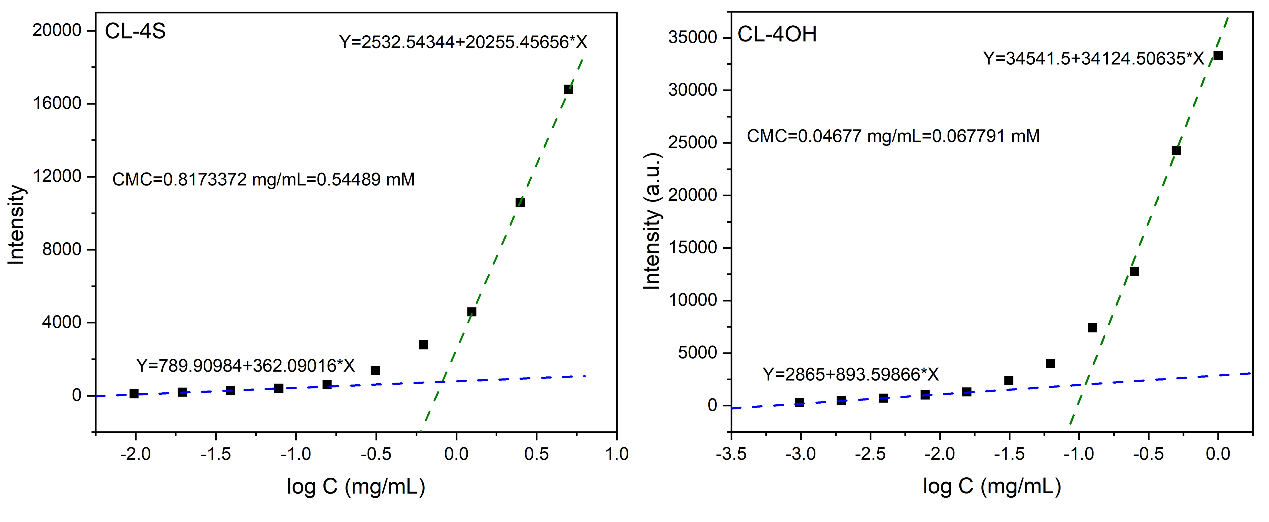


**Figure S11.** CMC determination of CL-4S surfactant in water by Nile red fluorescence intensity versus logarithmic CL-4S concentration. The CMC of CL-4S surfactant is 0.55 mM.


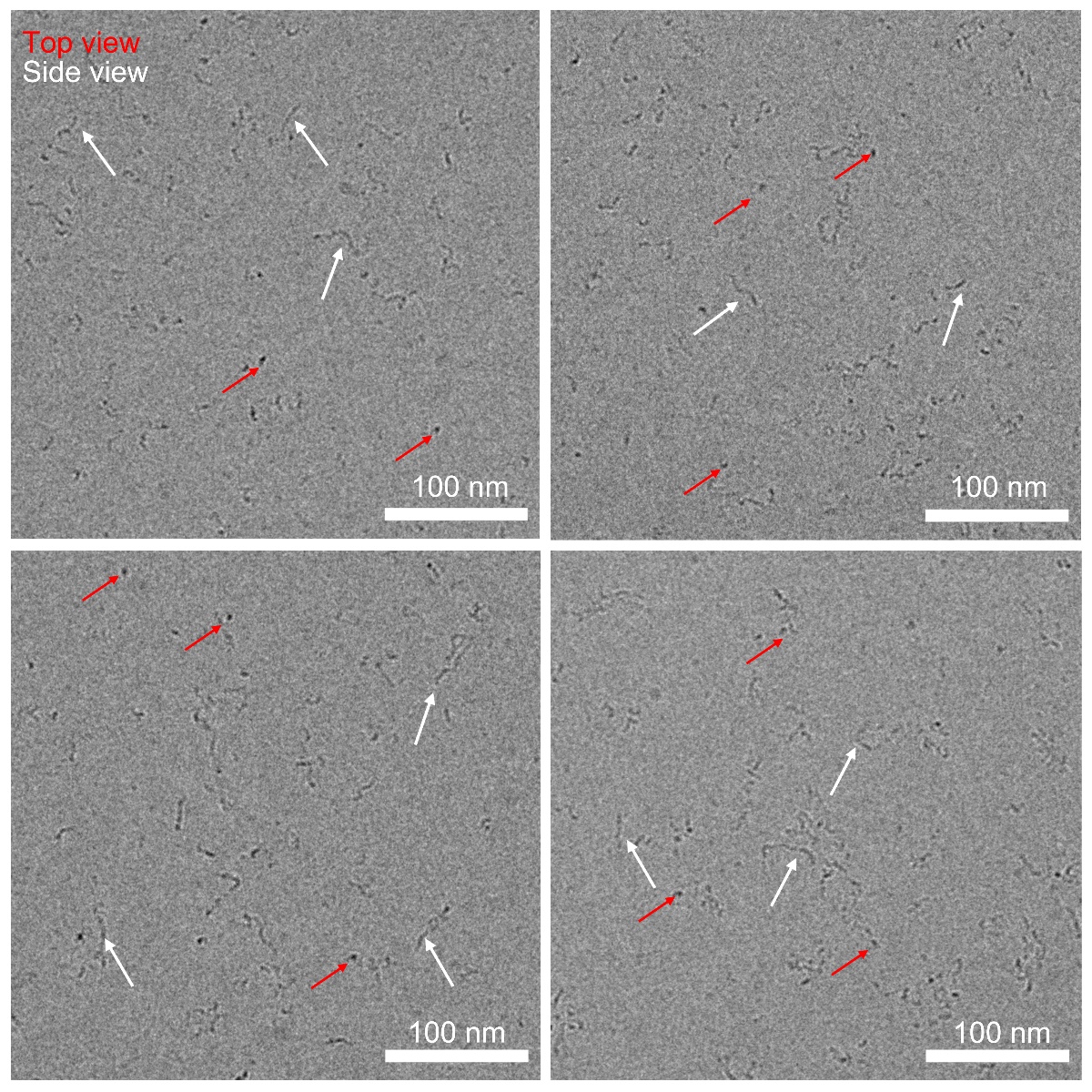


**Figure S12.** Cryo-TEM image of CL-4S surfactant in water from top and side views. Scale bar=100 nm.


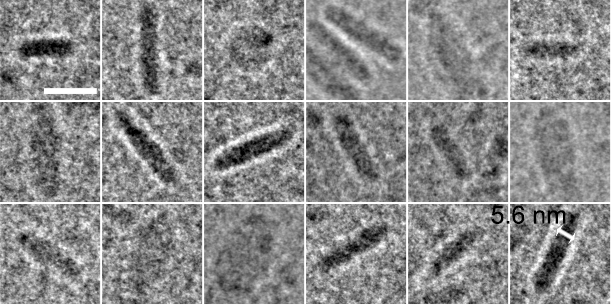


**Figure S13.** Cryo-TEM of 4S-Nanodiscs (DMPC/CL-4S/CL=8/1/1.33 mM). Scale bar=20 nm.


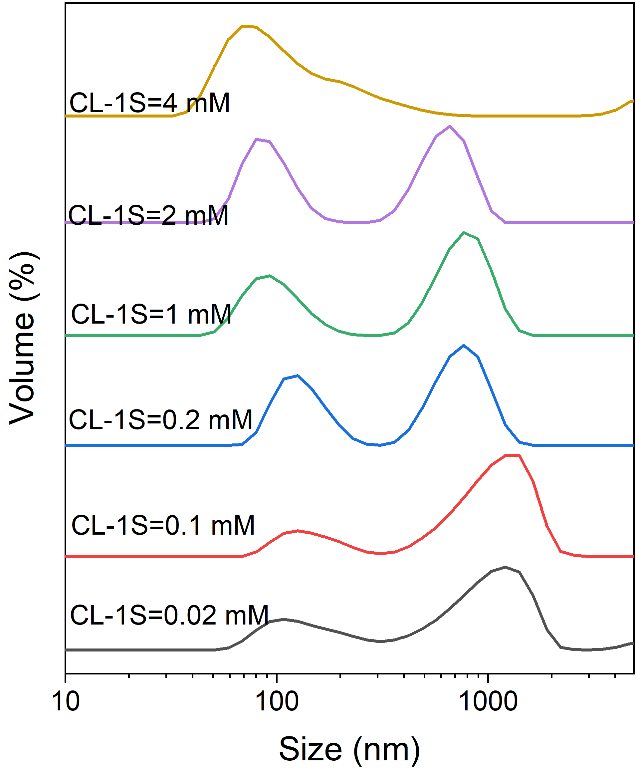


**Figure S14.** DLS profile of DMPC/CL with various concentrations of CL-1S.


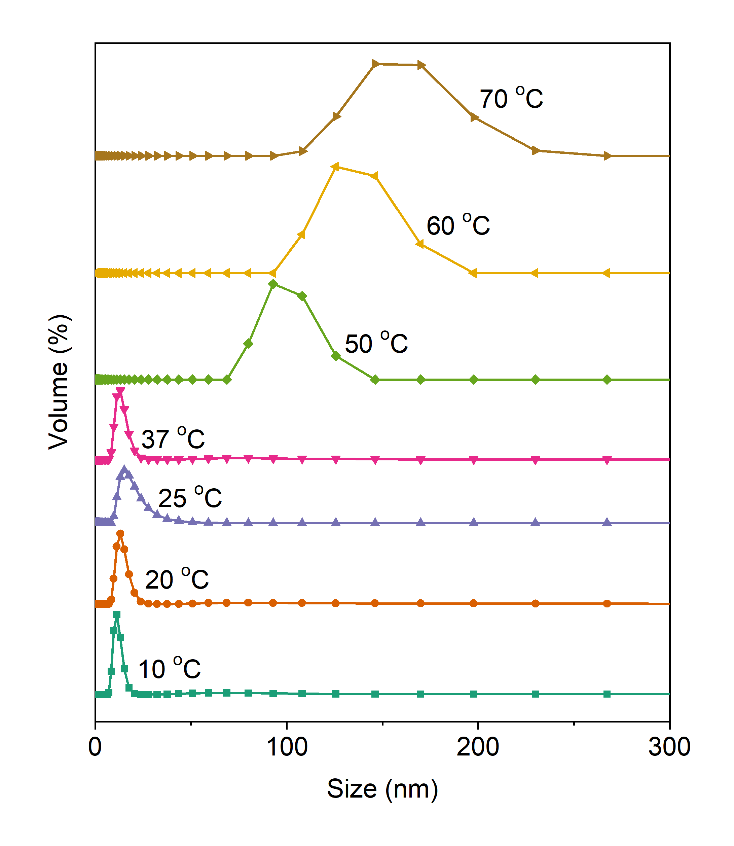


**Figure S15.** DLS profile of DMPC/CL with CL-4S under different temperature. DMPC/CL-4S/CL=8/1/1.33 mM.


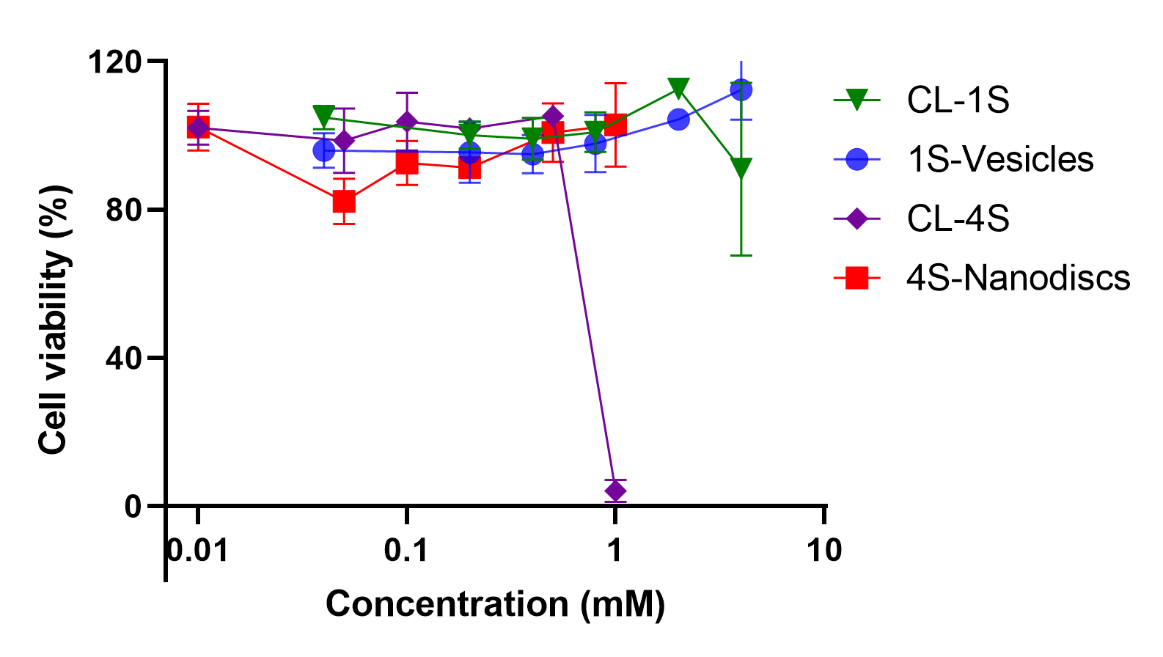


**Figure S16.** Evaluation of cellular toxicity of CL-1S, 1S-Vesicles, CL-4S, 4S-Nanodiscs incubated with Vero cells. Values are expressed as mean ±SD, n=3. The concentration in X-axis represents the concentration of CL-1S or CL-4S surfactants.


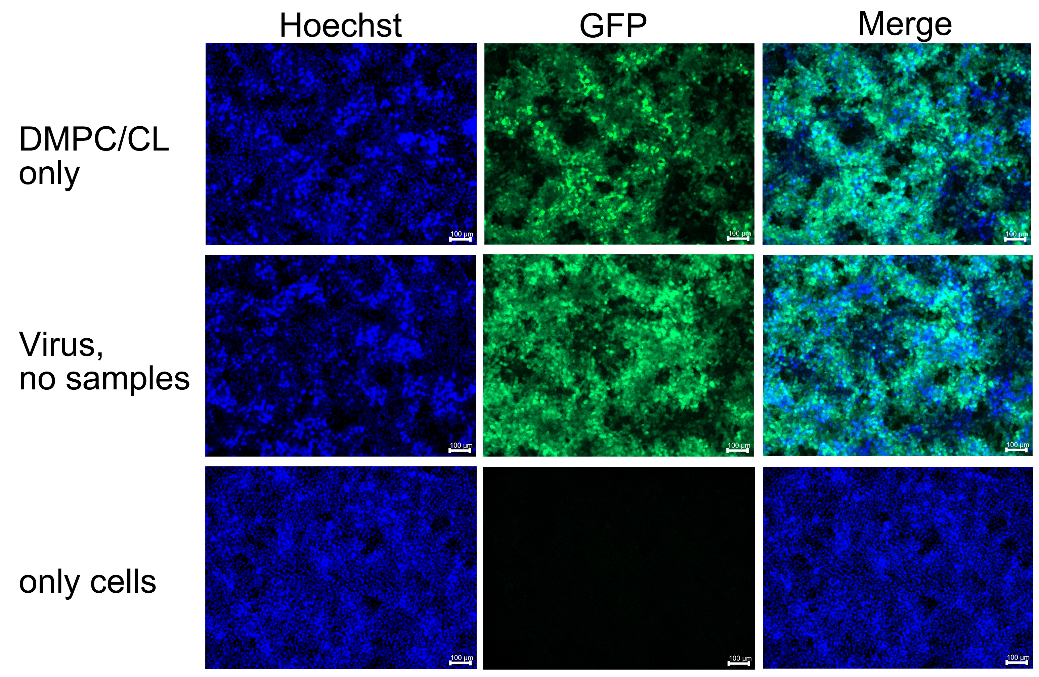


**Figure S17.** Immunofluorescent images for the uninfected cells, HSV-1-infected cells with and without DMPC/CL only (DMPC/CL=4/0.67 mM). Scale bar=100 µm.

# 3. Simulation Method and Results

## 3.1. Molecular dynamics (MD) simulations

To study the self-assembly behavior of the amphiphilic systems, we employed MD simulations using GROMACS.^[1]^ Atomistic simulations utilizing the CHARMM36 force field ^[2-3]^ were conducted to parameterize and map the structures to the coarse-grained (CG) level. CG simulations were performed using Martini 3 force field ^[4]^, in which the bead typing and validation of the force field are detailed in section 3.1. To facilitate the formation of stable nanostructures within accessible simulation timescales, surfactant concentrations were set higher than those used experimentally, in the range of 80-120 mM for total surfactants in water. Systems were constructed by randomly placing the surfactants (and Na+ counter ions in case of CL-1S and CL-4S containing systems to neutralize the total charge of the system) in water, resulting in a box size of approximately 530,000 beads (details of system components are listed in Table S1). These systems were initially energy minimized followed by a 1 ns NPT simulations with a time step of 5 fs. Production simulations were then conducted in the NPT ensemble at 310 K and 1 bar, using the V-rescale thermostat ^[5]^ and C-rescale barostat ^[6]^ with a 20 fs time step for at least 6 μs. Post-simulation analyses included visual inspection of self-assembled morphologies and measurement of bilayer thickness to evaluate structural features and molecular organization within the assemblies.

**Table S1.** Composition of the studied systems in MD simulations.

|  | DMPC /CL-1S  (8/1.33) | DMPC /CL-4S  (8/1.33) | DMPC/CL-4OH  (8/1.33) | DMPC/CL/CL-1S  (8/1.33/0.5) | DMPC/CL/CL-4S  (8/1.33/0.5) |
| --- | --- | --- | --- | --- | --- |
| DMPC | 3000 (80 mM) | 3000 (80 mM) | 3000 (80 mM) | 3000 (80 mM) | 3000 (80 mM) |
| CL | - | - | - | 500 (13.3 mM) | 500 (13.3 mM) |
| CL-1S | 500 (13.3 mM) | - | - | 188 (5 mM) | - |
| CL-4S | - | 500 (13.3 mM) | - | - | 188 (5 mM) |
| CL-4OH | - | - | 500 (13.3 mM) | - | - |
| Na ion | 500 (13.3 mM) | 2000 (53.3 mM) | - | 188 (5 mM) | 752 (20 mM) |
| CG water | 520781 | 520781 | 520781 | 520781 | 520781 |

### 3.1.1. Coarse-Grained Force Field Parameterization

The force field of DMPC and cholesterol were adopted from the previous parameterization of Borges-Araújo et al.^[7]^ Therefore, in this work, we just parameterize the modified cholesterol amphiphiles by modifying the pre-developed cholesterol force field and adjusting the parameters of the added parts of the molecules. To this end, the atomistic simulations using the CHARMM36 force field were first employed to obtain bonded parameter distributions for amphiphile components. Firstly, 100 amphiphiles were inserted in a box and solvated with water. Then, the boxes were energy minimized followed by 20 ns of NPT relaxations at 310 K and 1 bar controlled by V-rescale thermostat and C-rescale barostat. Bonded parameters were derived by mapping the last 5 ns of the atomistic trajectories to their corresponding CG representations shown in Figure S18.


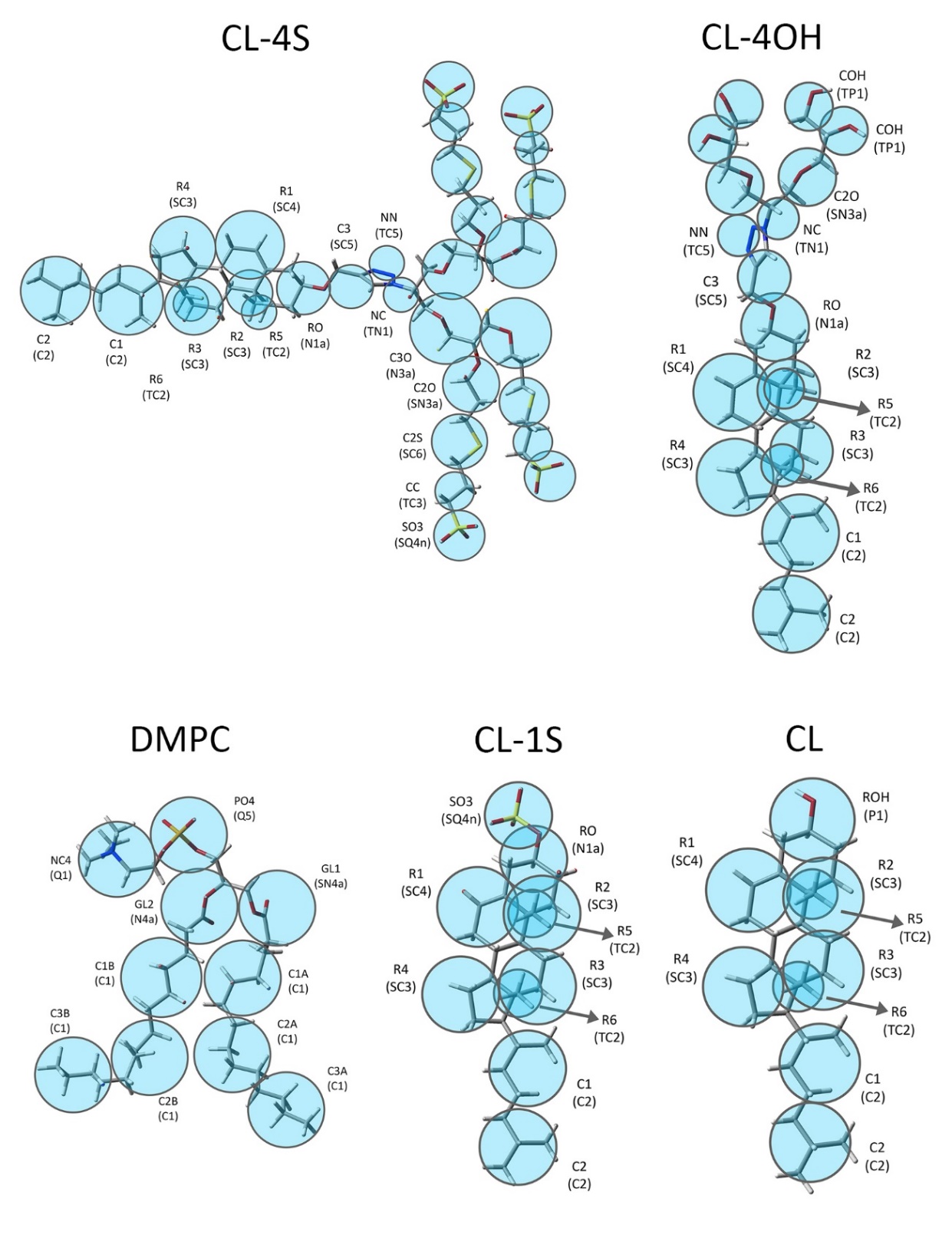


**Figure S18.** Martini CG model and bead types for components.

The distributions of the bonds and angles connecting the center of the geometry (COG) of the atoms of a bead were averaged over time. The distributions were converted into potential values via Boltzmann inversion (Equation S1). The resulting potential energy profiles were fit to harmonic functions (Equation S2, S3) to determine the CG force constants and equilibrium values. A list of equilibrium values and force constants for all bonded interactions at the CG level is provided in Table S2-S4. Some examples for the comparison of bonds and angles distribution for all-atom and CG levels are shown in Figure S19, exhibiting appropriate fitting of parameters.

$U=-ln\left( p \right)kT$ (S1)

$U_{bond}=\frac{1}{2}K_{bond}\left( l-l_{0} \right)^{2}$ (S2)

$U_{angle}=\frac{1}{2}K_{angle}\left( cos\left( \theta\right)-cos\left( \theta_{0} \right) \right)^{2}$ (S3)


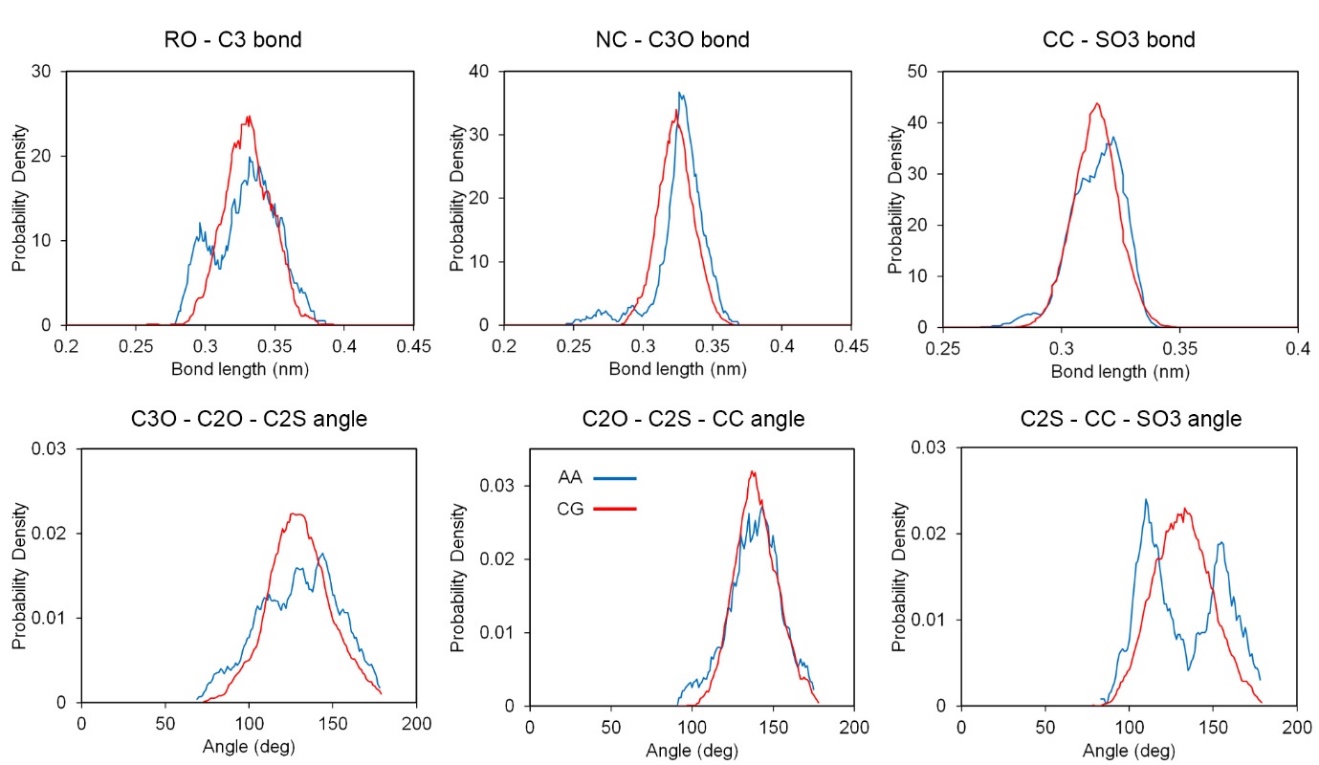


**Figure S19.** Some examples of all-atom and CG bond-length and bond-angle distribution.

**Table S2.** Bond and constraint parameters for CG structures.

|  | Bond | *l*_0_ (nm) | *K*_bond_ (kJ mol^-1^ nm^-2^) |
| --- | --- | --- | --- |
|  | DMPC | | |
|  | NC4-PO4 | 0.4 | 7000 |
|  | PO4-GL1 | 0.42 | 1350 |
|  | GL1-GL2 | 0.312 | 2500 |
|  | GL1-C1A | 0.47 | 5000 |
|  | C1A-C2A | 0.47 | 3800 |
|  | C2A-C3A | 0.47 | 3800 |
|  | C1B-C2B | 0.47 | 3800 |
|  | C2B-C3B | 0.47 | 3800 |
|  | GL2-C1B | 0.47 | 3600 |
|  | Cholesterol-based amphiphiles | | |
| C1-C2 | | 0.44 | 15000 |
| RO-SO3 | | 0.3 | 50000 |
| RO-C3 | | 0.33 | 10000 |
| C3-NN | | 0.26 | 40000 |
| C3-NC | | 0.36 | 40000 |
| NC-C3O | | 0.32 | 20000 |
| C3O-C2O | | 0.43 | 5000 |
| C2O-C2S | | 0.33 | 5000 |
| C2S-CC | | 0.32 | 5000 |
| CC-SO3 | | 0.31 | 50000 |
| NC-C2O | | 0.31 | 25000 |
| C2O-COH | | 0.32 | 12000 |
|  | COH-COH | 0.28 | 35000 |
|  |  |  |  |
|  | Constraint | *l*_0_ (nm) | |
|  | Cholesterol-based amphiphiles | | |
|  | C1-R1 | 0.75012 | |
|  | C1-R2 | 0.78504 | |
|  | R1-R2 | 0.34797 | |
|  | NN-NC | 0.275 | |

**Table S3.** Angles parameters for CG structures.

| Angle | *θ*_0_ (°) | *K*_angle_ (kJ mol^-1^ rad^-2^) |
| --- | --- | --- |
| DMPC | | |
| PO4-GL1-GL2 | 108.0 | 21.5 |
| PO4-GL1-C1A | 139.1 | 31.2 |
| GL1-C1A-C2A | 180.0 | 35 |
| C1A-C2A-C3A | 180.0 | 35 |
| GL2-C1B-C2B | 180.0 | 35 |
| C1B-C2B-C3B | 180.0 | 35 |
| Cholesterol-based amphiphiles | | |
| R4-R3-C2 | 99.0 | 250 |
| SO3-RO-R1 | 127.0 | 120 |
| SO3-RO-R2 | 144.5 | 300 |
| C3-RO-R1 | 115 | 180 |
| C3-RO-R2 | 142 | 300 |
| NN-NC-C3O | 95 | 230 |
| NC-C3O-C2O | 128 | 40 |
| C3O-NC-C3O | 120 | 200 |
| C3O-C2O-C2S | 130 | 30 |
| C2O-C3O-C2O | 68 | 440 |
| C2O-C2S-CC | 138 | 80 |
| C2S-CC-SO3 | 130 | 40 |
| NC-C2O-COH | 135 | 80 |
| C2O-COH-COH | 105 | 70 |

**Table S4.** Dihedral parameters for CG structures.

| Dihedral | *θ*_0_ (°) | *K*_dihedral_ (kJ mol^-1^) |
| --- | --- | --- |
| Cholesterol-based amphiphiles | | |
| R6-R4-R3-C2 | -70 | 50 |

Nonbonded interactions followed standard Martini 3 definitions based on bead polarity and type (see Figure S18).

### 3.1.2. Validation of CG Parameters

To validate the CG parameterization, we first simulated standard lipid bilayers composed of DMPC and cholesterol at different molar ratios (DMPC/CL = 6:1, 6:2, 6:3, 6:4) for 4-12 µs in the NPT ensemble at 310 K and 1 bar using the V-rescale thermostat and C-rescale barostat and a time step of 20 fs. The simulation boxes were constructed by randomly inserting 3000 DMPC molecules and the corresponding number of cholesterol molecules and solvating them in water. Visual and structural properties including self-assembly behavior, bilayer thickness, and vesicle size were obtained and compared to experimental and literature-reported values.


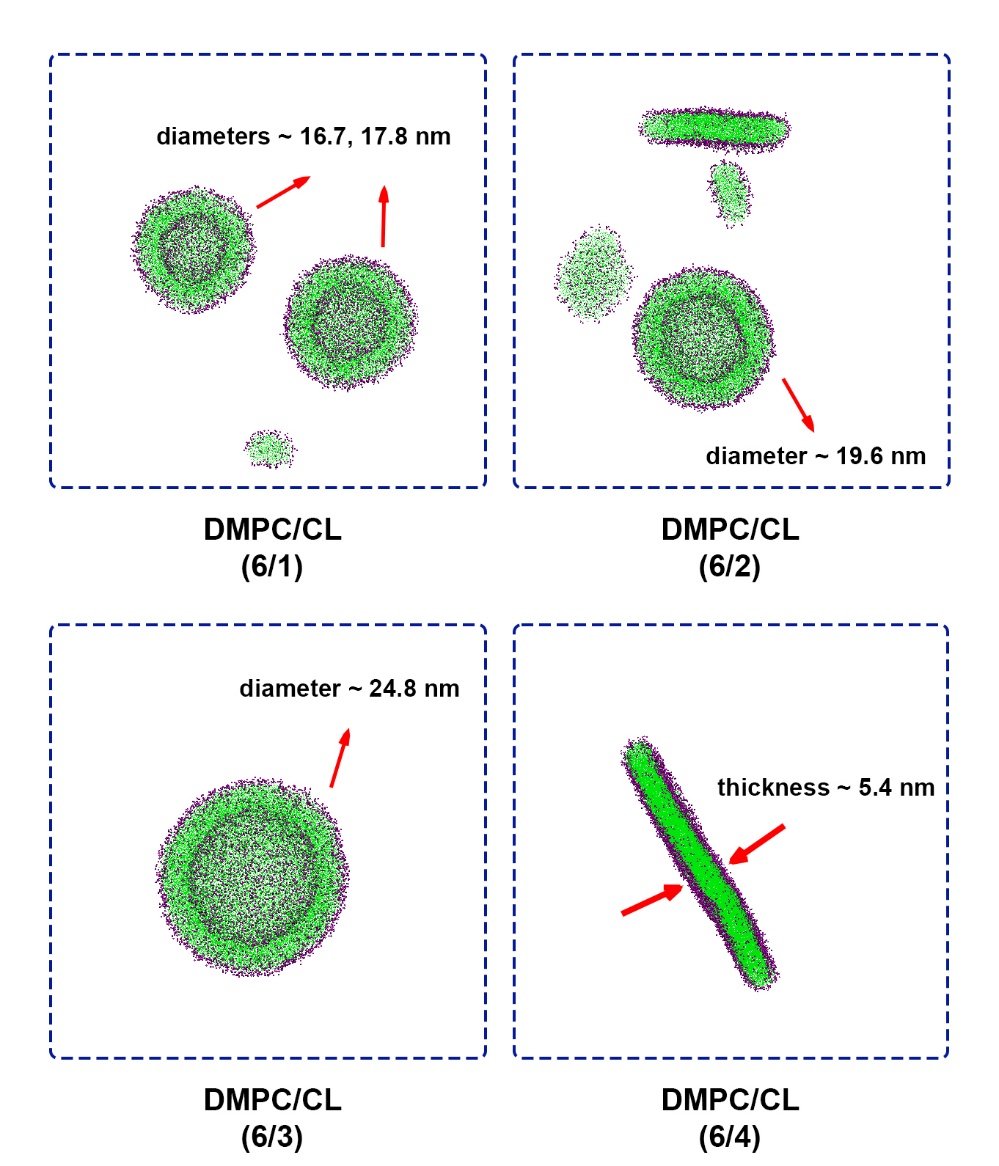


**Figure S20.** Representative CG snapshots of self-assembled DMPC/Cholesterol systems at various molar ratios.

As illustrated in Figure S20, all simulated systems exhibited bilayer formation with a consistent thickness of ~5.4 nm followed by bilayer curvature and forming vesicles, consistent with experimental expectations. Increasing the cholesterol content led to the formation of larger vesicles which corresponds well to previously reported behavior for DMPC-based bilayers ^[8-10]^. However, at the ratio of 6/4 of DMPC/CL, the increased rigidity of the lipid bilayer at higher cholesterol content resulted in stabilizing the system into elongated bilayer structure. As a result, a higher number of amphiphilic molecules would be required to overcome the bending stiffness and form vesicles. Therefore, under the current simulation conditions—specifically the system size and amphiphile count—vesicle formation cannot be captured. These results confirm that the CG force field accurately captures the expected structural assemblies.


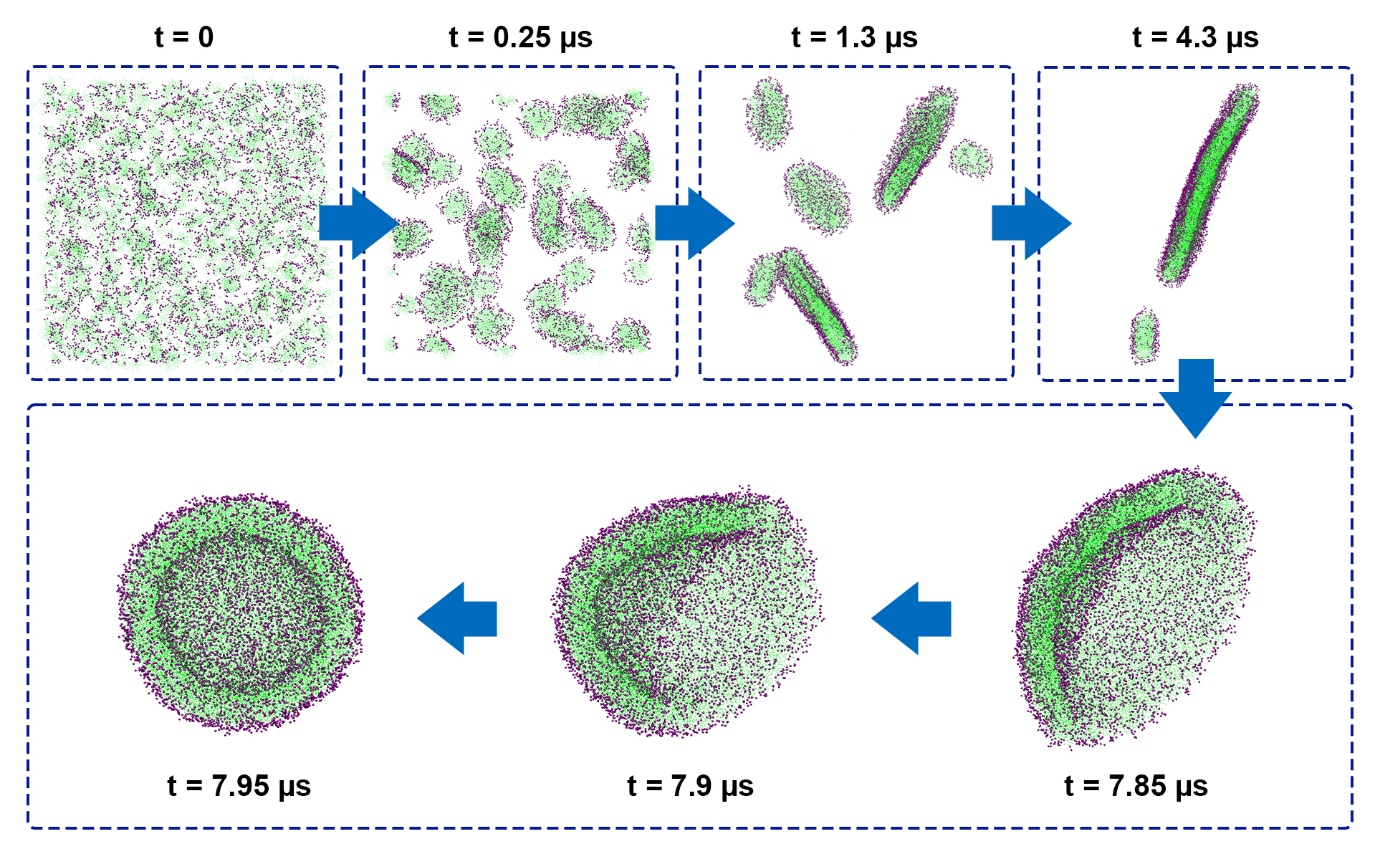


**Figure S21.** Representative CG snapshots of self-assembled DMPC/CL (6/3) system at various times.

Figure S21 illustrates the self-assembly process of DMPC/CL (6/3) system revealing the vesicle formation mechanism.


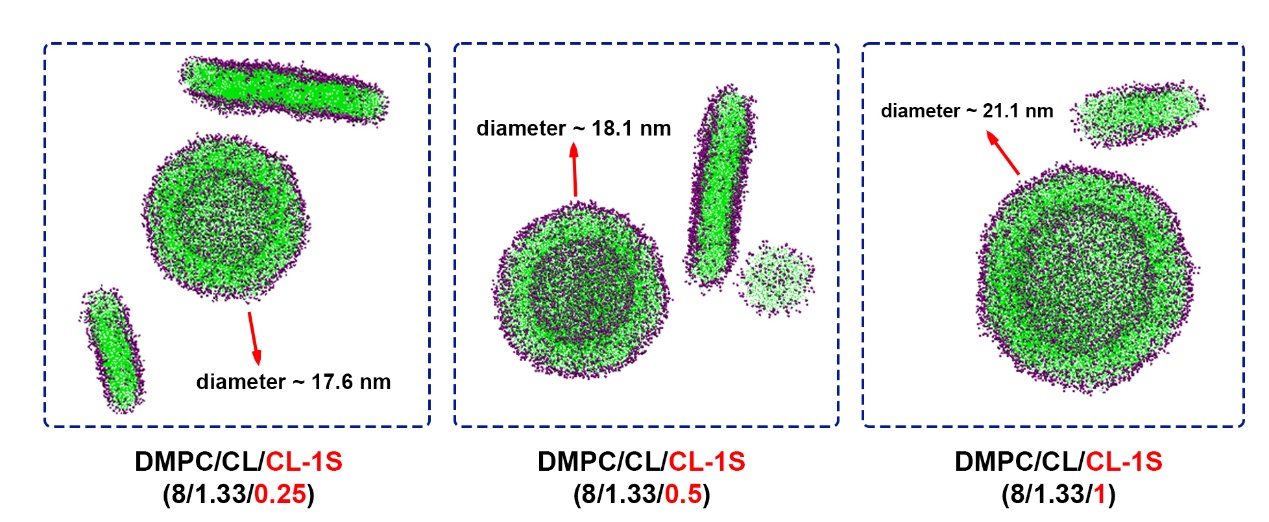


**Figure S22.** Morphology of self-assembled bilayer structures at different CL-1S contents in the simulation box after 4.5 µs.

## 3.2. 2D charge distribution on nanodiscs

This section explains the mean-field and Monte Carlo approaches employed to investigate the distribution of mobile charges in a 2D nanodiscs, and presents representative results derived from these methods.

### 3.2.1 System Hamiltonian

The system is modeled using the Hamiltonian for $N$ indistinguishable, interacting mobile particles with positions $r₁,...,r_{N}$:

$$H\left( r₁,...,r_{N} \right)=\left( 1/\beta\right)\int dr\mu\left( r \right)n\left( r \right)+\left( 1/2 \right)\int drdr'n\left( r \right)v\left( r-r' \right)n\left( r' \right)$$

where the number density profile is defined as:

$$n\left( r \right)=\sum_{i=1}^{N}\delta\left( r-r_{i} \right)$$

with $\mu\left( r \right)$ representing the generating field, used to calculate the density distribution, $v\left( r-r' \right)$ the pairwise interaction potential between particles at positions $r$ and $r'$, and $\beta=1/\left( k_{B}T \right)$ represents the inverse thermal energy.

### 3.2.2 Mean-Field Methodology

In the mean-field (MF) approach, we compute a variational free-energy functional derived from the Hamiltonian above, incorporating the Debye–Hückel interactions between particles and enforcing particle number conservation. From the mean-field self-consistent equation, the radial particle density $n\left( r \right)$ is computed iteratively, starting from a uniform initial distribution and an initial chemical potential $\mu=0$. To take advantage of the azimuthal symmetry inherent in the 2D disc geometry, the electrostatic potential is evaluated at each iteration as follows:

$$v\left( r \right)=\left( 2\pi q/\varepsilon\right)\left[ K₀\left( \kappa r \right)\int_{r₁}^{r}n\left( r' \right)I₀\left( \kappa r' \right)r'dr'+I₀\left( \kappa r \right)\int_{r}^{r₂}n\left( r' \right)K₀\left( \kappa r' \right)r'dr' \right]$$

where q is the particle charge, ε is the dielectric constant of water, $K₀$ and $I₀$ are modified Bessel functions, and $\kappa$ is the Debye screening parameter which is related to the salt concentration via $\kappa^{2}=8\pi l_{B}q^{2}c_{s}$. The density is updated using:

$$n\left( r \right)\Lambda^{3}=exp\left[ -\beta\int n\left( r' \right)v\left( r-r' \right)dr'-\mu\right]$$

with $\Lambda$ the thermal de Broglie wavelength and the chemical potential $\mu$ adjusted to satisfy the particle number conservation condition $\int n\left( r \right)dr=N₀$. The iteration continues until convergence is achieved, typically with a tolerance of $\|\Delta n{\|}_{\infty}<10⁻⁶$.

### 3.2.3 Monte Carlo Methodology

The Monte Carlo (MC) simulation employs the Metropolis–Hastings algorithm to sample particle configurations based on the Hamiltonian according to the Boltzmann distribution. Particles are confined to a 2D disc and interact via the Debye–Hückel potential:

$$v_{DH}\left( r \right)=l_{B}exp\left( -\kappa r \right)/r$$

where $l_{B}=e{^{2}}/\left( 4\pi\varepsilon k_{B}T \right)$ is the Bjerrum length. At each MC step, a particle is randomly selected and proposed to move by a small displacement. The energy change $\Delta E$ is computed using the Hamiltonian, and the move is accepted or rejected according to the Metropolis criterion with probability

$$min\left( 1,exp\left( -\beta\Delta E \right) \right)$$

The step size is adjusted during an equilibration phase (50,000,000 steps) to achieve an acceptance rate of approximately 40–50%. The total simulation runs for 100,000,000 steps, with configurations recorded to compute the mean radial density profile using 50 radial bins.

### 3.2.4 Monte Carlo Snapshots

To provide insight into the spatial arrangement of charges, we present a representative Monte Carlo snapshot for a charge valency $q=4$ , $N=20$ particles and an inverse screening length $\kappa=1.1nm⁻¹$, corresponding to a salt concentration of $\left( c_{S}=100 mM \right)$ on a 2D disc.


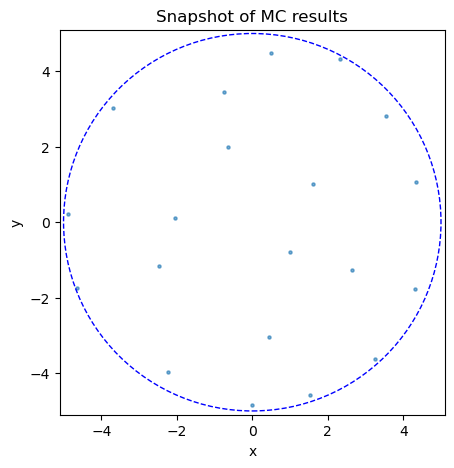


**Figure S23.** Monte Carlo snapshot showing the particle positions for $q=4,N=20,$ and $\kappa=1.1nm⁻¹\left( c_{S}=100 mM \right)$ on a 2D disc. Each dot represents a particle position.

### 3.2.5 Effect of Screening Parameter κ

The screening parameter $\kappa$ significantly influences the charge distribution. A larger $\kappa$ (stronger screening) results in a more uniform distribution, as electrostatic interactions are more effectively screened. A smaller $\kappa$ leads to pronounced charge accumulation near the disc's boundary due to reduced screening.


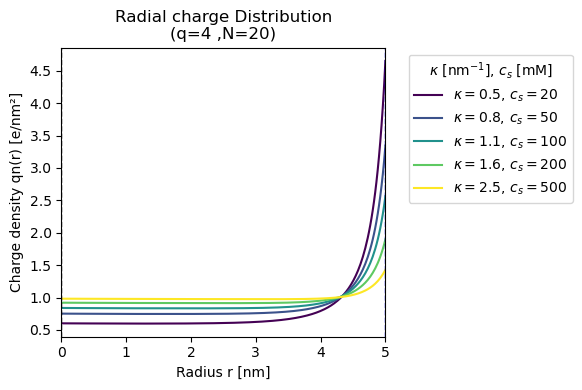


**Figure S24.** Mean-field theory results for the particle charge distribution for different $\kappa$ value.s $\left( \kappa=0.5, 0.8, 1.1, 1.6, 2.5 nm⁻¹ \right)$ with $q=4$ and $N=20$.


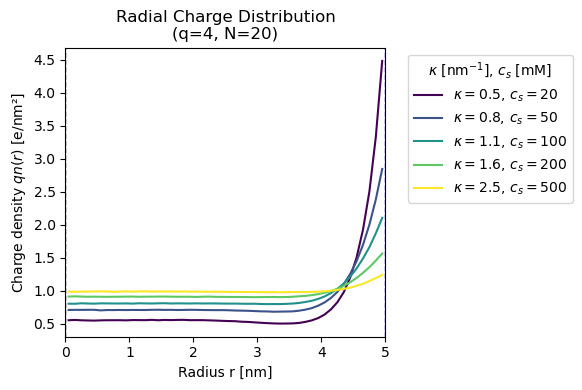


**Figure S25.** Monte Carlo simulation results for the particle charge distribution for different $\kappa$ values $\left( \kappa=0.5, 0.8, 1.1, 1.6, 2.5 nm⁻¹ \right)$with $q=4$ and $N=20$.

# 4. Supporting Videos

**Video S1.** Green and purple colous represent the hydrophobic and hydrophilic parts of the amphiphiles, respectively. Besides, the water molecules are removed from the simulation box for visualization enhancement in the video.

**References:**

[1] B. H. Paul Bauer, & Erik Lindahl., *Zenodo* **2023**.

[2] S. Jo, T. Kim, V. G. Iyer, W. Im, *J. Comput. Chem.* **2008**, *29*, 1859-1865.

[3] J. Lee, X. Cheng, S. Jo, A. D. MacKerell, Jr., J. B. Klauda, W. Im, *Biophys. J.* **2016**, *110*, 641a.

[4] P. C. T. Souza, R. Alessandri, J. Barnoud, S. Thallmair, I. Faustino, F. Grünewald, I. Patmanidis, H. Abdizadeh, B. M. H. Bruininks, T. A. Wassenaar, P. C. Kroon, J. Melcr, V. Nieto, V. Corradi, H. M. Khan, J. Domański, M. Javanainen, H. Martinez-Seara, N. Reuter, R. B. Best, I. Vattulainen, L. Monticelli, X. Periole, D. P. Tieleman, A. H. de Vries, S. J. Marrink, *Nat. Methods* **2021**, *18*, 382-388.

[5] G. Bussi, D. Donadio, M. Parrinello, *J. Chem. Phys.* **2007**, *126*, 014101.

[6] M. Bernetti, G. Bussi, *J. Chem. Phys.* **2020**, *153*, 114107.

[7] L. Borges-Araújo, A. C. Borges-Araújo, T. N. Ozturk, D. P. Ramirez-Echemendia, B. Fábián, T. S. Carpenter, S. Thallmair, J. Barnoud, H. I. Ingólfsson, G. Hummer, D. P. Tieleman, S. J. Marrink, P. C. T. Souza, M. N. Melo, *J. Chem. Theory Comput.* **2023**, *19*, 7387-7404.

[8] S. Chakraborty, M. Doktorova, T. R. Molugu, F. A. Heberle, H. L. Scott, B. Dzikovski, M. Nagao, L.-R. Stingaciu, R. F. Standaert, F. N. Barrera, J. Katsaras, G. Khelashvili, M. F. Brown, R. Ashkar, *Proc. Natl. Acad. Sci.* **2020**, *117*, 21896-21905.

[9] A. Bañuelos-Frias, V. M. Castañeda-Montiel, E. R. Alvizo-Paez, E. A. Vazquez-Martinez, E. Gomez, J. Ruiz-Garcia, *Sec. Soft Matter Physics* **2021**, *Volume 9 - 2021*.

[10] J. Peters, J. Marion, F. J. Becher, M. Trapp, T. Gutberlet, D. J. Bicout, T. Heimburg, *Sci. Rep.* **2017**, *7*, 15339.
